# Preceding Infections and Coagulation Biomarkers in Early-Onset Cryptogenic Ischemic Stroke

**DOI:** 10.1161/STROKEAHA.125.052134

**Published:** 2026-03-17

**Authors:** Barbara M. Hulsen, Janneke P. Spiegelenberg, Nicolas Martinez-Majander, Lauri Tulkki, Tomi Sarkanen, Pekka Jäkälä, Petra Redfors, Juha Huhtakangas, Pauli Ylikotila, Bettina von Sarnowski, Nilufer Yesilot, Ulrike Waje-Andreassen, Ana Catarina Fonseca, Patricia Martínez-Sánchez, Janika Kõrv, Phillip Ferdinand, Kristina Ryliskiene, Alessandro Pezzini, Radim Licenik, Marialuisa Zedde, Juha Sinisalo, Eva Gerdts, Tuukka A. Helin, Lotta Joutsi-Korhonen, Tímea Szántó, Frederick Palm, Armin J. Grau, Frank-Erik de Leeuw, Jukka Putaala

**Affiliations:** Department of Neurology (B.M.H., N.M.-M., L.T., J.P.), Helsinki University Hospital and University of Helsinki, Finland.; Department of Cardiology, Heart and Lung Center (J.S.), Helsinki University Hospital and University of Helsinki, Finland.; Department of Clinical Chemistry, HUS Diagnostic Center (T.A.H., L.J.-K.), Helsinki University Hospital and University of Helsinki, Finland.; Department of Internal Medicine, the Netherlands (B.M.H., J.P.S.).; Department of functional coagulation, Synapse Research Institute, the Netherlands (J.P.S.).; Department of Neurology, Tampere University Hospital, Wellbeing Services County of Pirkanmaa, and Faculty of Medicine and Health Technology, Tampere University, Finland (T. Sarkanen).; Neurocenter Neurology, Kuopio University Hospital, Finland (P.J.).; University of Eastern Finland (P.J.).; Department of Neurology, Sahlgrenska University Hospital and Department of Clinical Neuroscience, Institute of Neuroscience and Physiology, Sahlgrenska Academy at University of Gothenburg, Sweden (P.R.).; Department of Neurology, Oulu University Hospital and University of Oulu, Finland (J.H.).; Department of Neurology, Turku University Hospital and University of Turku, Finland (P.Y.).; Department of Neurology, University Medicine Greifswald, Germany (B.v.S.).; Department of Neurology, Istanbul University, Istanbul Faculty of Medicine, Turkey (N.Y.).; Department of Neurology, Haukeland University Hospital, Bergen, Norway (U.W.-A.).; Department of Neurology, Hospital de Santa Maria, Faculty of Medicine, University of Lisbon, Portugal (A.C.F.).; Department of Neurology, Torrecardenas University Hospital, University of Almería, Spain (P.M.-S.).; Department of Neurology and Neurosurgery, University of Tartu, Estonia (J.K.).; Neurosciences, University Hospitals of North Midlands NHS Trust, Stoke-on-Trent, UK (P.F.).; Vilnius University, Faculty of Medicine, Center of Neurology, Lithuania (K.R.).; Department of Medicine and Surgery, University of Parma and Stroke Care Program, Department of Emergency, Parma University Hospital, Italy (A.P.).; North West Anglia NHS Foundation Trust, Acute Stroke Centre, United Kingdom (R.L.).; Neurology Unit, Stroke Unit, Azienda Unità Sanitaria Locale-IRCCS Reggio Emilia, Italy (M.Z.).; Center for Research on Cardiac Disease in Women, Department of Clinical Science, University of Bergen, Norway (E.G.).; Coagulation Disorders Unit, Department of Hematology, Comprehensive Cancer Centre, Comprehensive Care Centre of Hemophilia and Allied Disorders (EAHAD), Helsinki University Hospital, Finland (T. Szántó).; Department of Neurology, Klinikum Ludwigshafen, Germany (F.P., A.J.G.).; Department of Neurology, Donders Institute for Brain, Cognition and Behaviour, Centre for Neuroscience, Radboud University Medical Center, The Netherlands (F.-E.d.L.).

**Keywords:** biomarkers, blood coagulation, hemostasis, infection, ischemic stroke, risk factors, von Willebrand Factor

## Abstract

**BACKGROUND:**

Infections might transiently trigger ischemic stroke through thromboinflammatory mechanisms, which may be especially relevant in young patients with cryptogenic ischemic stroke (CIS). This study assessed the association between infections, their characteristics, and coagulation biomarkers in young patients with CIS.

**METHODS:**

The SECRETO study is a multicenter case-control study at 19 European centers (2013–2022), enrolling first-ever patients with CIS aged 18 to 49 years and age- and sex-matched controls. Self-reported preceding infections within 3 months were assessed using a standardized questionnaire, and blood samples were collected at baseline and 3-month follow-up. The primary outcome was the association between preceding infections and CIS, analyzed using conditional logistic regression adjusted for demographic and vascular risk factors. Secondary outcomes were coagulation biomarkers (VWF [von Willebrand Factor], FVIII [factor VIII], fibrinogen, antithrombin III, and protein C) in relation to infection characteristics.

**RESULTS:**

Among 537 matched pairs, infections in the preceding week were associated with 2.6-fold higher CIS odds (odds ratio, 2.64 [95% CI, 1.34–5.20]) after multivariable adjustment. VWF activity was higher in cases than in controls (122 IU/mL versus 100 IU/mL; *P*<0.001). Within cases only, VWF activity was higher in cases with recent infections compared to those without infection (157 IU/mL versus 121 IU/mL). In controls, VWF levels did not differ by infection status. In stratified case-control analyses, each SD increase in VWF and factor VIII was linked to higher stroke odds in participants with recent infections or fever.

**CONCLUSIONS:**

This multicenter case-control study shows that recent infections are associated with higher odds of early onset CIS. Stratified analyses indicate that VWF and factor VIII are more strongly associated with CIS in the presence of recent infections. Future studies should further elucidate infection-related thromboinflammatory mechanisms underlying CIS, including a potential increased sensitivity to inflammatory triggers, and explore whether preventive measures could reduce risk in this population.

**REGISTRATION:**

URL: https://www.clinicaltrials.gov; Unique identifier: NCT01934725.

The incidence of ischemic stroke in the young population aged between 18 and 49 years has increased over the past years.^1,2^ This rise cannot solely be explained by traditional risk factors or causes, as the number of cryptogenic ischemic stroke (CIS) cases is particularly increasing.^3^ CIS is more prevalent in young patients,^4^ representing a substantial portion of early onset strokes.^5^ Understanding its pathogenesis is crucial for developing more effective secondary prevention, especially given the debilitating consequences of the stroke.^6^

Apart from chronic vascular risk factors, temporary triggers, occurring shortly before the event, have been proposed to increase ischemic stroke risk.^7^ Preceding infections have been coined potential transient triggers.^8,9^ Interestingly, studies have shown that the risk of stroke after an infection is higher in younger compared to older individuals.^9–12^ Only 1 study has specifically explored this association in young patients with CIS.^13^

Studies in young patients have found minimal differences in the prevalence of traditional or nontraditional vascular risk factors between those with and without preceding infections,^14,15^, supporting the hypothesis that infections may independently trigger early-onset ischemic stroke. In older adults, a shorter interval between infection and stroke increases the risk of stroke, peaking in the week preceding stroke.^9,10,16–19^ However, the specific characteristics of infections that heighten stroke risk remain largely unexplored in younger populations with distinct risk factor profiles compared to older adults.

The exact mechanisms of infection leading to stroke are still unclear. One prevailing hypothesis, known as thromboinflammation, suggests the interaction of infections with the hemostatic system.^8^ Invasion of pathogens may activate various stages of blood clot formation through complex interplay with the immune system, eventually resulting in thrombotic diseases.^20^ This mechanism may be particularly relevant in early onset CIS cases,^8,21^ where conventional vascular risk factors are often absent, and infections may play a relatively larger role. However, the hematologic mechanisms underlying the link between infection and stroke have not been thoroughly studied, especially in early-onset stroke.^8,22,23^

We conducted a case-control study to investigate the association between characteristics of preceding infections, coagulation biomarkers, and the risk of early-onset CIS.

## Methods

### Data Availability Statement

Data are available on reasonable request. The SECRETO study (Searching for Explanations for Cryptogenic Stroke in the Young, Revealing the Etiology, Triggers and Outcome) was approved by the Ethics Committee of the Hospital District of Helsinki and Uusimaa; all recruiting centers have obtained approvals from the responsible local Ethics Committees.

### Study Population and Design

Young patients aged 18 to 49 years with CIS and age- and sex-matched stroke-free controls were enrolled between November 2013 and January 2022 at 19 European centers participating in the prospective multicenter SECRETO study. Cases aged 18 to 49 years with a first-ever ischemic stroke of unknown cause as defined by the TOAST (Trial of ORG 10172 in Acute Stroke Treatment) criteria,^24^ confirmed by brain magnetic resonance imaging, were included after providing written informed consent. A standardized protocol was followed to exclude known causes of ischemic stroke, as previously described.^25^ Patent foramen ovale was classified as high-risk if accompanied by an atrial septal aneurysm or a large shunt. The severity of stroke was assessed using the National Institutes of Health Stroke Scale (NIHSS) score.

For each case, 1 stroke-free control was prospectively recruited from the same study center, preferably within 3 months of the corresponding case. Cases and controls were matched by sex, region, and maximum age difference of 5 years, which was treated as exact matching. When feasible, controls were selected through population registry searches, using mailing lists generated without prior knowledge of participants’ willingness to take part. Individuals were approached one-by-one, and they were not part of any predefined pool of potential study participants. Otherwise, cases’ unrelated proxies (eg, friends or partners) were invited. We excluded case-control pairs without data on preceding infections.

Medical history and questionnaire data were collected from cases as soon as possible after stroke, at the time of consent and enrollment. Median time from stroke to inclusion was 6 days. Blood samples were obtained concurrently with questionnaire completion at baseline, and again at a 3-month follow-up visit. Controls attended a single study visit during which a detailed medical history was taken, questionnaires were completed, and blood samples were collected. There was no follow-up blood sample taken from controls.

The study is reported to adhere to the STROBE checklist (Strengthening the Reporting of Observational Studies in Epidemiology) for case-control studies (Supplemental Material).^26^

### Cardiovascular Risk Factors and Comorbidities

A detailed medical history was taken from the participants in-person by a study doctor or nurse, for cases during index hospitalization and for controls at the study visit. Demographic variables and cardiovascular risk factors were reported, including age, level of education, sex, history of cardiovascular disease, diabetes, hypertension, hypercholesterolemia, current smoking, obesity, history of any chronic multisystem disorder, venous thrombosis, and malignancy, migraine with aura, current illicit drug use, and current estrogen use. See Table S1 for definitions of these variables.

### Preceding Infections

Self-reported preceding infections within 3 months before the stroke or study visit, and their characteristics were assessed using a questionnaire, consisting of 13 questions for cases and 12 for stroke-free controls (Figure S1). Preceding infection was defined as any symptoms of infection in the past 3 months, further specified by timing, mode (acute or chronic), locus, pathogen (bacterial or viral), treatment, symptoms, and fever. Infection mode was classified as chronic if the duration exceeded 4 weeks, and as acute otherwise. Recent infection was defined as within a week, and remote infection as within 8 to 90 days preceding stroke or study visit. Confirmed infections required verification by a physician or laboratory results. Participants reported episodes of elevated body temperature in the past 2 weeks, categorized as 37.5 °C to 38.5 °C or above 38.5 °C. Fever was defined as a body temperature exceeding 37.5 °C.

### Coagulation Biomarkers

In cases, blood samples were collected at baseline (within 2 weeks following the stroke) and at 3-month follow-up. In controls, blood samples were only collected during the baseline study visit. Samples were processed following a standardized protocol and transferred to the Biobank Core Lab at the Institute for Molecular Medicine Finland, University of Helsinki, for storage (−80 °C) and analyzed at HUSLAB, Helsinki, Finland. For the present study, sodium citrated plasma was used to measure fibrinogen (g/L) and activity levels of VWF (von Willebrand Factor, IU/mL), coagulation FVIII (factor VIII, %), AT3 (antithrombin III, %), and PC (protein C, %; Supplemental Methods). CRP (C-reactive protein) levels (mg/L), as a surrogate marker of systemic inflammation, were measured in cases on admission at their respective centers.

### Statistical Analysis

Baseline characteristics and biomarker levels were compared between matched cases and controls using the McNemar test for proportions and the Wilcoxon signed-rank test for non-normally distributed continuous variables. Differences in infection characteristics were assessed among participants with a preceding infection. As not all matched pairs contained both a case and a control with a preceding infection, unmatched comparisons of infection characteristics between cases and controls were analyzed using the Pearson χ^2^ test for proportions and the Wilcoxon Rank-Sum test for non-normally distributed continuous variables. Normality was assessed through visual inspection of histograms.

Conditional logistic regression models for matched case-control analysis were used to analyze the association between infection characteristics and CIS, yielding unadjusted and adjusted odds ratios (ORs) with corresponding 95% CIs. Preselected covariates for the adjusted model included age, education level, hypertension, diabetes, hypercholesterolemia, history of cardiovascular disease, and current smoking, as these are well-documented stroke risk factors, and might also influence the infection characteristics.

Within-group analyses were performed separately in cases and controls, comparing baseline biomarker levels across infection characteristics identified in earlier analyses. Furthermore, biomarker levels in cases were compared between baseline and 3-month follow-up to explore temporal changes. Nonparametric tests for non-normally distributed continuous data were used, including the Kruskal-Wallis test for unpaired data with more than 2 levels, followed by the Mann-Whitney *U* test for pairwise comparisons if significant.

Stratified binary logistic regression analyses were performed within strata defined by the presence or absence of infection characteristics to investigate baseline levels of biomarkers that showed potential associations with both CIS and infection characteristics. Within each separate model, adjusted ORs for CIS odds were calculated per SD increase in biomarker levels.

Additional analyses within the matched case-control design were performed to assess the robustness and generalizability of the findings. To assess the impact of the COVID-19 pandemic, infection characteristics were compared between participants enrolled before January 2020. Seasonal influences were analyzed by comparing monthly enrollment and infection rates. The association between baseline coagulation biomarkers and the interval from infection or stroke to blood sample was assessed using scatterplots. Scatterplots were also used to assess the correlation between NIHSS score and baseline biomarker levels. A multivariate conditional logistic regression model, excluding cases classified as likely atherothrombotic according to revised TOAST criteria,^27^ was used to assess whether the associations persisted in this group.

In addition, case-only descriptive analyses were conducted to provide complementary context. Demographics, vascular risk factors, and stroke severity were compared across 3 separate analyses: (1) cases with versus without preceding infection; (2) cases with recent infection (1–7 days) versus cases with a remote or no infection; and (3) infection characteristics in relation to the presence of a likely atherothrombotic cause. The influence of high-risk patent foramen ovale was assessed by comparing infection characteristics between cases with or without a high-risk patent foramen ovale.

IBM SPSS Statistics for Windows, V.29.0 (IBM) and R version 4.4.0 were used for analysis. Two-sided hypothesis tests were performed, with *P*<0.05 considered significant.

## Results

### Study Participants and Demographics

Of the initial 546 case-control pairs, 537 young cases with first-ever CIS (median age, 41 [interquartile range, 34–46]; 47.3% female) and 537 matched stroke-free controls were included. Nine matched pairs were excluded (Table S2) due to missing data on preceding infections. In cases, information was obtained from proxies in only 9 individuals (1.7%). Out of 537 controls, 313 (58.3%) were recruited from population registries, while the remaining 224 were recruited from the social network of the patient. Compared with controls, cases more often had a lower level of education and were more likely to have risk factors, including hypertension, current smoking, obesity, any chronic multisystem disorder, a history of venous thrombosis, migraine with aura, and estrogen use (Table 1). Of all cases, 14% could be classified as likely atherothrombotic, characterized by the presence of multiple traditional risk factors (≥3) but without clear evidence of atherosclerosis.^27^

**Table 1.**
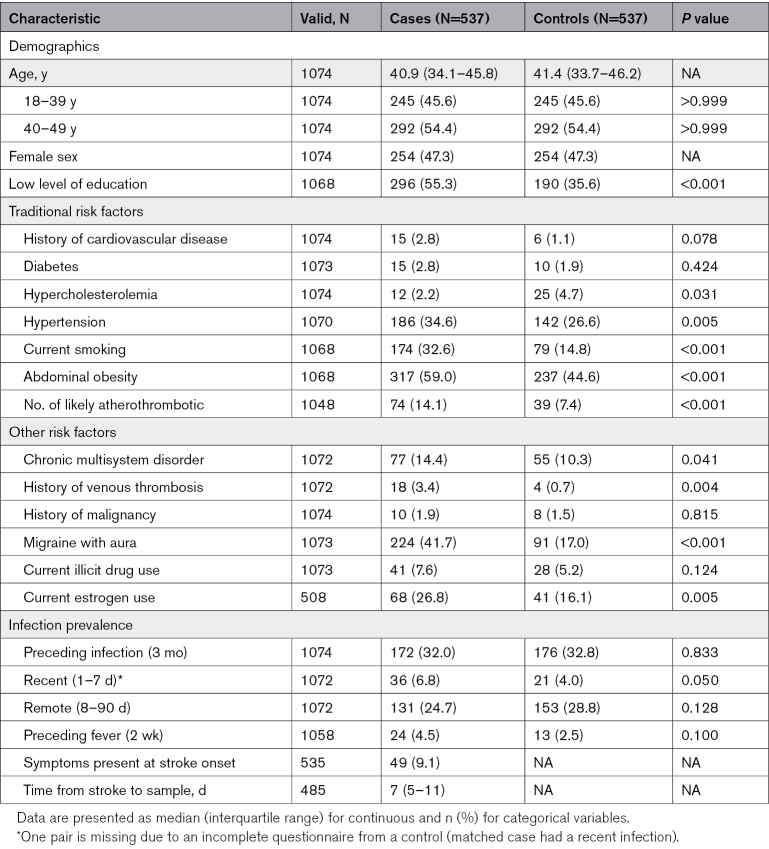
Demographics, Comorbidities, Preceding Infection Prevalence of the Study Population

### Prevalence of Preceding Infections

No significant difference was observed in the proportion of participants reporting an infection within the 3 months preceding stroke or baseline visit (32.0% in cases versus 32.8% in controls; *P*=0.833). However, cases more often reported an infection in the preceding week compared to controls (6.8% versus 4.0%; *P*=0.050). There was no significant difference in preceding fever within the past 2 weeks (4.5% versus 2.5%; *P*=0.100; Table 1).

### Infection Characteristics

Table 2 presents infection characteristics among cases and controls who reported a preceding infection in the last 3 months. In cases, infections were more often within 1 week preceding the stroke, whereas in controls, infections were more remote (recent infections, 22.0% versus 12.1%; *P*=0.014). Infections in cases were more often confirmed by a physician or laboratory tests (39.0% versus 23.9%; *P*=0.002). The majority of infections were acute, viral, and involved the respiratory tract in both cases and controls. However, compared to controls, cases had a higher proportion of chronic (lasting >4 weeks; 9.1% versus 3.4%; *P*=0.030), bacterial (22.1% versus 11.7%; *P*=0.015), and nonrespiratory infections (25.5% versus 11.1%; *P*<0.001).

**Table 2.**
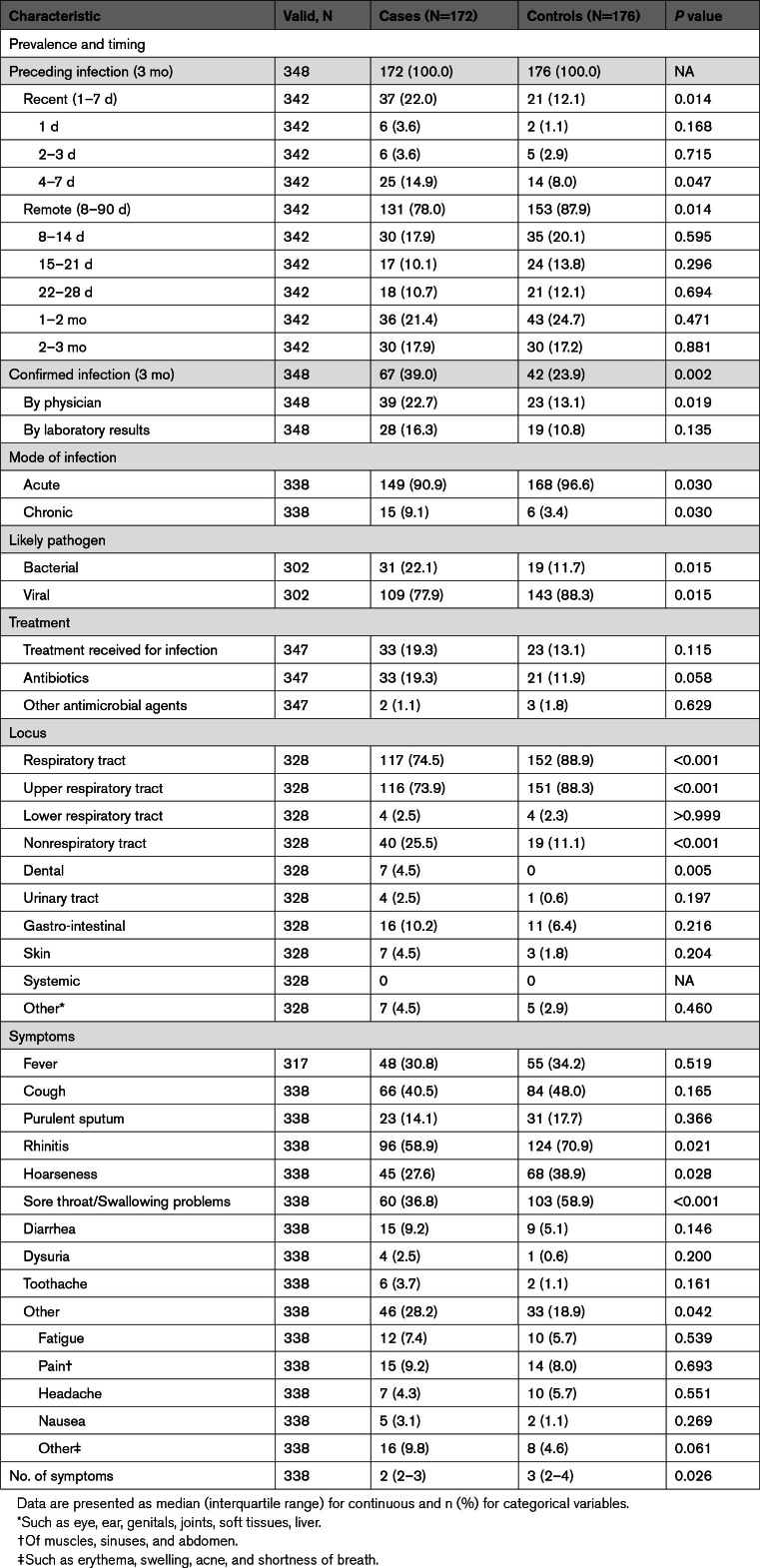
Detailed Characteristics of Preceding Infections in the Study Population

### Association Between Infection Characteristics and Early Onset CIS

**Figure 1.**
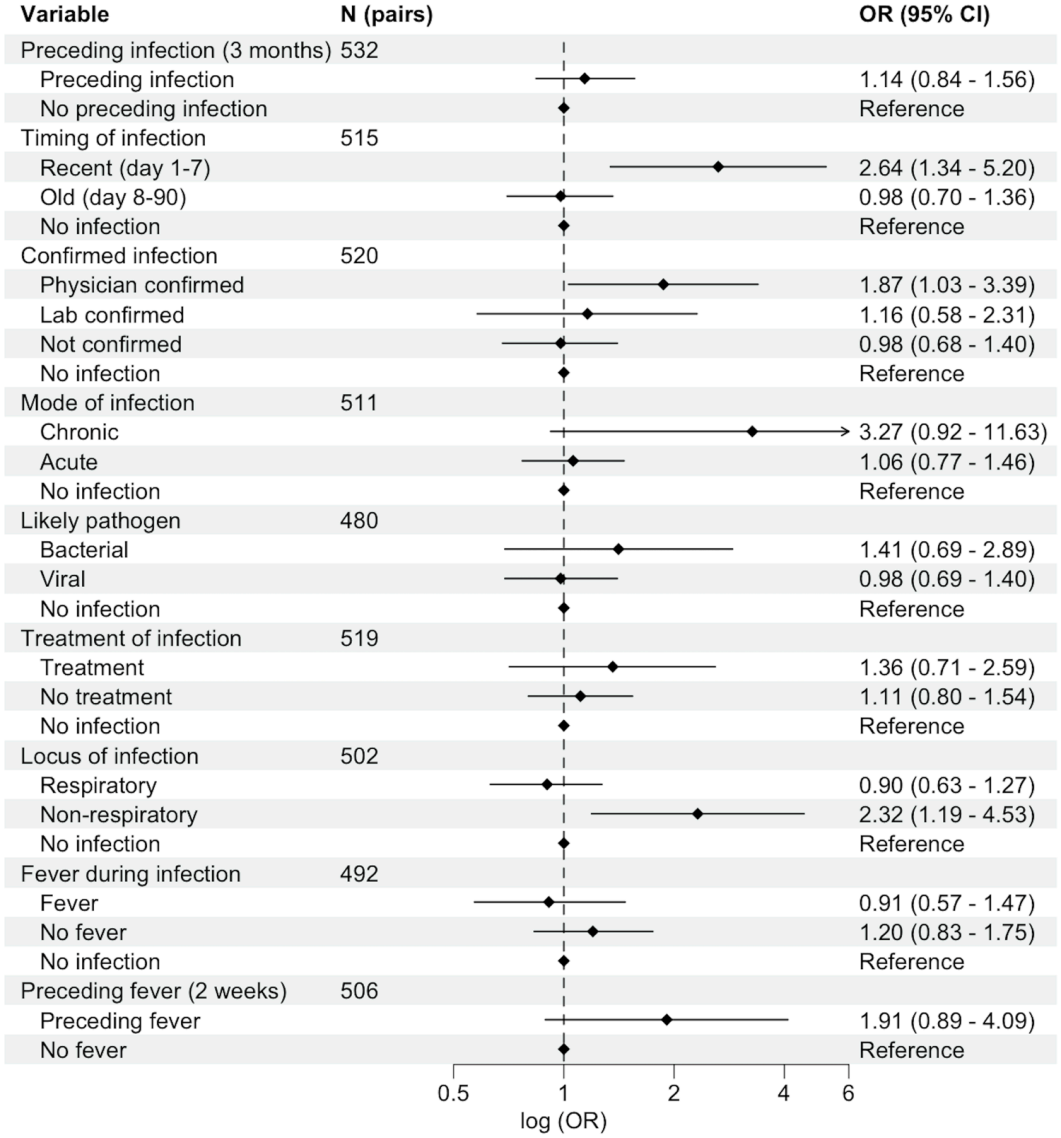
**Complete adjusted conditional logistic regression results for infection characteristics and odds of early onset cryptogenic ischemic stroke.** Data are presented as odds ratios (ORs) and 95% CI. Results are based on 9 separate conditional logistic regression models, each adjusted for age, education level, hypertension, diabetes, hypercholesterolemia, history of cardiovascular disease, and current smoking. Pairs with missing data were excluded from the models. The dotted line denotes the reference line. Chronic denotes duration >4 weeks; N (pairs), number of case-control pairs included in each model.

Primary results of the conditional logistic regression models can be found in Figure 1. Recent infections (in the preceding week) were associated with 2.6-fold higher odds of CIS after adjustment (OR, 2.64 [95% CI, 1.34–5.20]). Preceding infections within 3 months were associated with a significantly higher odds of CIS when confirmed by a physician (OR, 1.87 [95% CI, 1.03–3.39]) or classified as nonrespiratory infections (OR, 2.32 [95% CI, 1.19–4.53]). Preceding fever within 2 weeks was not associated with CIS. Unadjusted ORs can be found in Figure S2.

### Coagulation Biomarkers and Infection Characteristics

**Figure 2.**
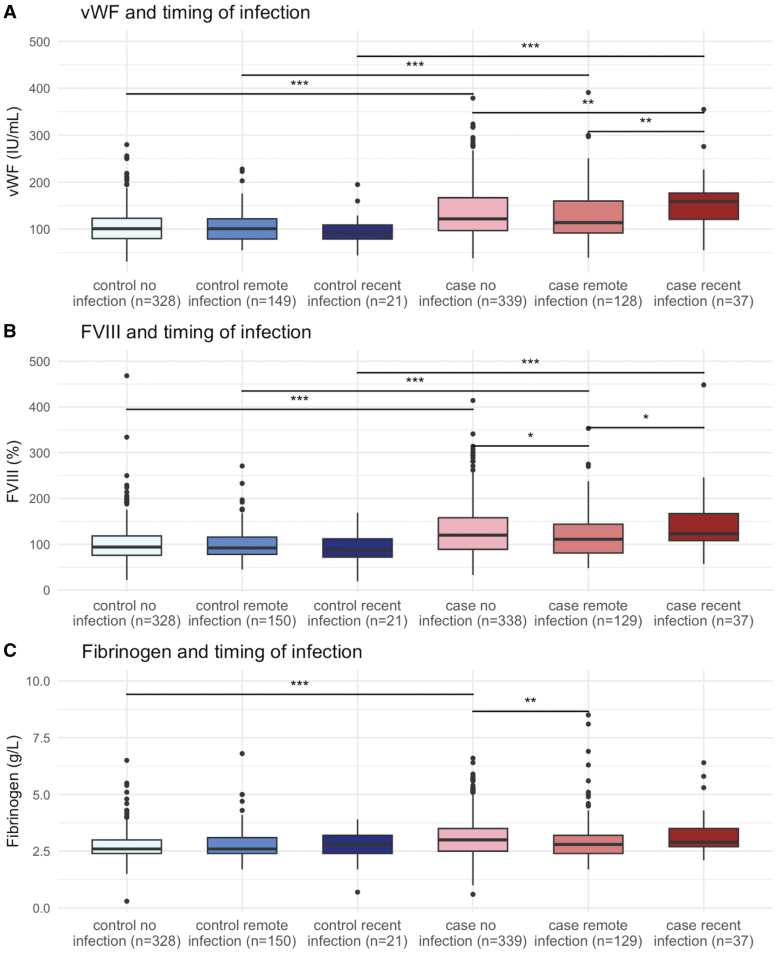
**Distribution of VWF (von Willebrand Factor), FVIII (factor VIII), and fibrinogen by timing of preceding infection in patients with cryptogenic ischemic stroke and stroke-free controls.** Data are presented as median, with interquartile ranges, and CIs. Recent denotes within 1–7 days before stroke/study visit; remote, within 8 to 90 days before stroke/study visit; pat, patient with cryptogenic ischemic stroke; and ctrl, stroke-free control. *P<0.05, **P<0.01, ***P<0.001. Only significant comparisons shown.

All coagulation biomarkers were significantly higher in cases compared to stroke-free controls at baseline (Table S3). Within-group analyses, no significant differences in coagulation biomarkers were found between cases with a preceding infection in the past 3 months compared with those with no infection (Figure S3). However, in cases with a recent infection, VWF was significantly higher (157 IU/mL) compared with cases with a remote (114 IU/mL, *P*=0.001) infection, or without a preceding infection (121 IU/mL, *P*=0.006; Figure 2). Among controls, VWF levels did not differ significantly across infection subgroups. Admission CRP followed the same pattern (Figure S4). FVIII was significantly higher in cases with a recent infection (123%) or no infection (122%), compared with cases with a remote infection (110%). Fibrinogen levels were significantly lower in cases with a remote infection, compared to cases without an infection. Similar differences were not observed in stroke-free controls (Figure 2).

Within-group analyses revealed no differences in AT3 and PC levels across any of the infection characteristics (Figures S5 and S6).

In cases only, a procoagulant shift in coagulation biomarkers emerged in the presence of preceding fever. Fibrinogen, VWF, and FVIII seemed elevated, while AT3 seemed lower in cases with a fever above 38.5 °C compared with those with a lower body temperature (Figure S7). However, these differences were not statistically significant.

Three months after CIS, all biomarker levels significantly decreased from their baseline levels, and almost all differences across infection characteristics decreased or diminished (Figures S8 through S10).

### Coagulation Biomarkers and CIS Odds, Stratified by Infection Characteristics

**Figure 3.**
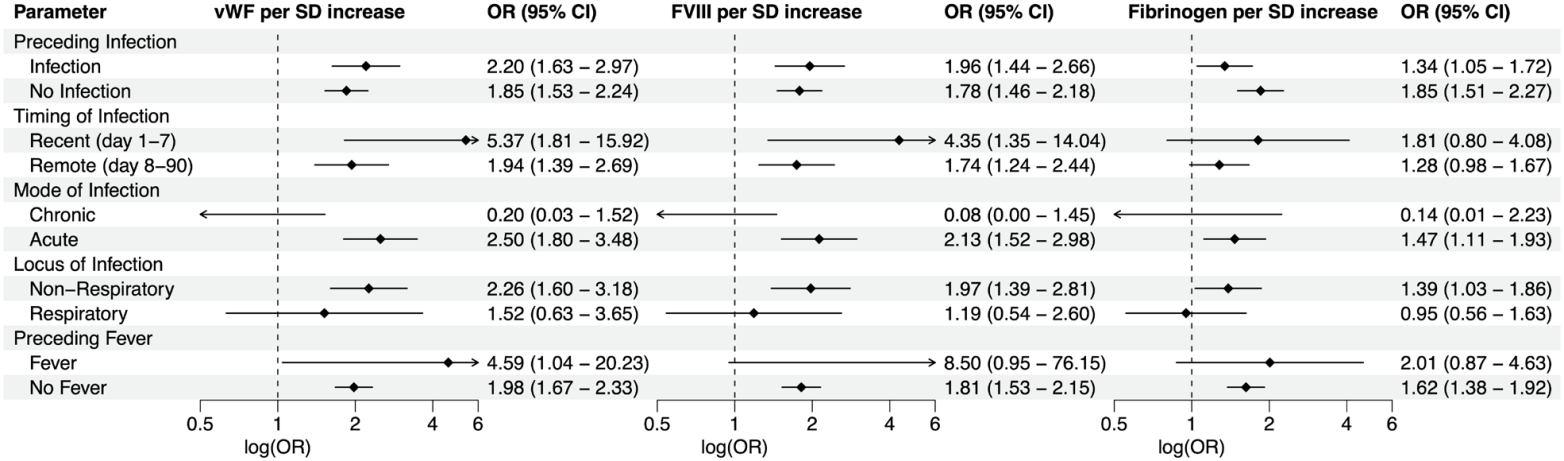
**Adjusted odds ratios and 95% CIs of VWF (von Willebrand Factor), coagulation FVIII (factor VIII), and fibrinogen per SD increase, and cryptogenic ischemic stroke odds stratified by infection characteristics.** Data are shown as odds ratios (ORs) and 95% CI. The dotted line denotes the reference line. Preceding infection, any infection in the past 3 months; preceding fever, body temperature >37.5 °C in the past 2 weeks; chronic, duration >4 weeks.

In stratified case-control analyses, each SD increase in VWF, FVIII, and fibrinogen was associated with substantially higher odds of CIS in participants with recent infections, compared to those with remote or no infections (Figure 3). These increased odds were also more pronounced in cases with acute infections than in those with chronic infections (lasting >4 weeks). Fever was also associated with a markedly elevated CIS odds per SD increase in VWF and FVIII. Additionally, all 3 biomarkers significantly increased CIS odds in nonrespiratory infections, while no significant associations were observed in respiratory infections (Figure 3). Unadjusted ORs can be found in Figure S11.

### Additional Analyses

Within the matched case-control design, COVID-19 sensitivity analysis showed similar frequencies of infection characteristics in participants recruited before 2020 (data not shown). Cases and controls showed similar patterns in the month of inclusion and prevalence of preceding infections (Figure S12). Baseline biomarker levels were not significantly affected by the interval from infection or stroke to blood sample (Figure S13) or by NIHSS scores (Figure S14). ORs for infection characteristics remained similar when excluding likely atherothrombotic cases (Figure S15).

In case-only descriptive analyses, cases without preceding infection were more likely to be current smokers and had a higher prevalence of stroke with a likely atherothrombotic origin (Table S4). Cases with a recent infection were more likely to have a history of venous thrombosis (Table S5). A higher proportion of likely atherothrombotic cases was found in participants with a chronic versus an acute infection (Table S6). No differences in infection parameters were observed between cases with and without high-risk patent foramen ovale (data not shown).

## Discussion

This multicenter matched case-control study is the first to investigate the association between infection characteristics, coagulation biomarkers, and early onset CIS. Our results demonstrated that recent infections in the preceding week were associated with 2.6-fold higher odds of early onset CIS. Within cases, VWF levels were higher in cases with recent infections compared to cases without infection, while this difference was not observed in stroke-free controls. A stratified case-control analysis further revealed that the association between VWF and FVIII with CIS was stronger in those with a recent infection. Finally, we also showed that fever in the 2 weeks preceding stroke appeared to influence all the biomarkers investigated in cases only, elevating VWF, FVIII, fibrinogen, and PC and decreasing AT3.

Our results suggest that only recent infections increase the odds of early-onset CIS. These findings align with previous evidence showing that the infection-stroke association strengthens as the time between infection and ischemic stroke shortens,^8,28^ with the strongest associations found within 1 week.^28^ The relationship between preceding infections and stroke is further supported by increased odds of CIS after confirmed infections. A new hypothesis posits that rather than individual infections, the cumulative effect of multiple infections, or infectious burden, could increase stroke risk.^29^ Future research should explore whether this holds for young patients with CIS, as our study lacked this data. Additionally, screening serum, urine, and sputum for pathogens linked to early onset CIS, and exploring the protective role of vaccinations would be warranted.

Fibrinogen, VWF, and FVIII are all thought to be involved in thromboinflammatory pathways, a complex interplay between coagulation and inflammation, that may explain the activation of coagulation after infectious triggers.^30,31^ However, previous studies reported inconclusive results on biomarkers that could potentially influence the pathway from infection to stroke.^19,32–34^ A recent review highlighted vasculopathies associated with viral infections, including endothelial damage, leading to endothelial activation.^35^ On activation, endothelial cells release VWF^8^ and FVIII, leading to a procoagulant state through several mechanisms, including platelet recruitment and eventually thrombin generation.^30,31,36,37^ Viral infections may also elevate thrombin levels by increasing tissue factor concentration,^35^ promoting thrombosis. Moreover, experimental studies have shown that VWF and FVIII rapidly rise after injection with bacterial toxins, suggesting that pathogens interact with endothelial cells and platelets, resulting in a procoagulant state.^38,39^ Fibrinogen is also recognized as an acute-phase reactant, and therefore, rises in response to infection and endothelial damage.^40^ Taken together, this evidence suggests that fibrinogen, VWF, and FVIII are likely to be elevated in stroke patients with a recent infection.

In accordance, our within-group analyses found that VWF was elevated in cases with recent (1–7 days) infections, while this difference was not observed in stroke-free controls. These findings could suggest that patients with cryptogenic stroke may exhibit a more pronounced response to infection contributing to stroke onset. This heightened response may reflect an underlying vulnerability or an increased sensitivity to an inflammatory stimulus.^41^ Furthermore, this elevation was transient and not sustained at the 3-month follow-up. CRP followed a similar pattern at baseline, suggesting that inflammatory pathways are involved in the elevated stroke risk after recent infections. Additionally, stratified case-control analysis showed that the association between VWF and CIS was stronger in participants with a recent or acute infection, as opposed to remote (8–90 days) or chronic (duration of >4 weeks) infections. Together, this further supports the hypothesis of recent infections triggering thromboinflammatory cascades, with VWF playing a pivotal role. Moreover, FVIII and fibrinogen appear to influence the risk of CIS based on infection characteristics, such as recent and acute infections, suggesting there may also be a role for FVIII and fibrinogen in these pathways. Importantly, biomarker levels did not vary according to NIHSS scores, indicating that these changes were not merely a consequence of stroke severity.

In a previous case-crossover study involving young stroke patients (aged 18 to 49 years), fever in the preceding 24 hours was associated with a 7.9-fold increase in the odds of CIS.^13^ In contrast, this case-control study found no direct association with fever. However, there was a shift observed in VWF, FVIII, fibrinogen, and AT3 toward a procoagulant state in only cases with preceding fever. In the stratified case-control analysis, VWF and FVIII were associated with substantially higher CIS odds in participants with preceding fever compared to those without. Notably, this study is the first to compare biomarkers in patients with stroke with and without fever before stroke, indicating a need for further research to clarify these relationships.

The study’s main strengths are a well-defined cohort of young patients with CIS that underwent a standardized, extensive diagnostic work-up, which provides relevant insights into a population particularly susceptible to infection-related stroke. Nearly all cases had biomarker data available from the 3-month follow-up, allowing evaluation of whether the observed differences at baseline were transient. The available data on confirmed infections made the observed association more reliable. The standardized diagnostic protocol minimized variability, ensuring accurate diagnosis and improving validity. Consecutive enrollment of cases minimized selection bias. Moreover, the case-control design reduces recall bias, improving the accuracy of exposure data compared to a case-crossover design. Finally, centralized analysis of biomarkers ensured consistency and comparability across centers.

Several limitations of the study are also recognized. Although this study is generalizable to young patients with CIS in the European centers, its applicability to other populations is limited. Factors such as postponing visits due to severe infections and seasonal variations may have influenced infection prevalence in controls. Furthermore, the use of both population-based controls and controls recruited from patients’ social networks may have introduced different sources of confounding and selection bias. Controls recruited via patients’ proxies may share exposures with cases, whereas population-based controls may differ in socio-demographic characteristics. Moreover, subgroup analyses were underpowered due to small sample sizes, particularly for preceding fever and chronic infections. For the same reason, we could not adjust for all possible confounders in the conditional logistic regression models, which may have resulted in residual confounding. Additionally, blood samples were taken within 2 weeks after stroke symptoms, so they may not have captured the full extent of biomarker changes, as coagulation factors can be elevated due to acute phase reactions and may fluctuate significantly during the acute phase of stroke. However, NIHSS scores did not differ between patients with CIS with and without preceding infection, suggesting that stroke severity did not influence the observed associations. An important limitation of case-control studies is recall bias. In our study, cases were asked to complete the questionnaire as soon as possible after the event or baseline study visit to limit inaccuracies. Nonetheless, recall bias may still have affected both cases and controls, particularly regarding remote infections, while it is less likely to have substantially affected information on recent infections, which were the main focus of our analyses. Finally, we could only assess associations, since case-control studies cannot establish causality.

To conclude, our study showed a significant association between recent infections and the increased odds of CIS in young patients. Stratified case-control analyses showed that higher VWF and FVIII levels were more strongly associated with CIS in those with a recent infection versus no infection. These findings suggest that patients with CIS may exhibit a stronger thromboinflammatory response to infection, possibly reflecting increased sensitivity to inflammatory triggers. Given the distinct profiles of young patients compared to older populations, future research should focus on elucidating the underlying thromboinflammatory mechanisms and exploring potential preventive measures that could mitigate this risk in younger individuals.

## ARTICLE INFORMATION

### Acknowledgments

B.M. Hulsen, Dr Spiegelenberg, and Dr Putaala designed the study, analyzed the data, and prepared the first version of the article. All authors contributed to acquiring the data, critically reviewed and edited the article, and approved the final version of the article. Dr Putaala had full access to all the data in the study and takes responsibility for the integrity of the data and the accuracy of the data analysis.

### Sources of Funding

### Disclosures

Dr Spiegelenberg was an employee of Synapse Research Institute, part of the Stago Diagnostica Group. The other authors report no conflicts.

### Supplemental Material

Supplemental Methods

Tables S1–S6

Figures S1–S15

STROBE Checklist

## Supplementary Material


