# Supplementary material for: Preceding Infections and Coagulation Biomarkers in Early-Onset Cryptogenic Ischemic Stroke
Source: Stroke. 2026 Mar 17;57(5):1220–30. doi: 10.1161/STROKEAHA.125.052134 (PMC13117575; doi:10.1161/STROKEAHA.125.052134)
Supplement: Supplementary file 1 [file str-57-1220-s001.pdf]

SUPPLEMENTARY MATERIALS

Preceding infections and coagulation biomarkers in early-onset cryptogenic ischemic stroke

Authors: Barbara Hulsen[1,2]; Janneke Spiegelenberg[2,3]; Nicolas Martinez-Majander[1]; Lauri Tulkki[1]; Tomi Sarkanen[4]; Pekka Jäkälä[5]; Petra Redfors[6]; Juha Huhtakangas[7]; Pauli Ylikotila[8]; Bettina von Sarnowski[9]; Nilufer Yesilot[10]; Ulrike Waje-Andreassen[11]; Ana Catarina Fonseca[12]; Patricia Martinez Sanchez[13]; Janika Kõrv[14]; Phillip Ferdinand[15]; Kristina Ryliskiene[16]; Alessandro Pezzini[17]; Radim Licenik[18]; Marialuisa Zedde[19]; Juha Sinisalo[20]; Eva Gerdts[21]; Tuukka A. Helin[22]; Lotta Joutsi-Korhonen[22]; Tímea Szántó[23]; Frederick Palm[24]; Armin J Grau[24]; Frank-Erik de Leeuw[25]; Jukka Putaala[1]

Contents:

Supplemental methods.
Figure S1. Questionnaire on preceding infections for cases and controls.
Figure S2. Unadjusted odds ratios (OR) and their confidence intervals (CI) for infection parameters and risk of early-onset cryptogenic ischemic stroke.
Figure S3. Distribution of von Willebrand Factor, Factor VIII and fibrinogen by preceding infection and mode of infection.
Figure S4. Distribution of C-reactive protein by preceding infection and mode of infection.
Figure S5. Antithrombin III (AT3) levels across infection parameters.
Figure S6. Protein C (PC) levels across infection parameters.
Figure S7. Distribution of von Willebrand Factor, Factor VIII, fibrinogen and antithrombin III by level of preceding fever in the preceding two weeks in cases only.
Figure S8. Von Willebrand Factor levels across infection parameters after three months.
Figure S9. Coagulation factor VIII levels across infection parameters after three months.
Figure S10. Fibrinogen levels across infection parameters after three months.
Figure S11. Unadjusted odds ratios and 95% confidence intervals of von Willebrand Factor, coagulation factor VIII and fibrinogen per standard deviation increase, and cryptogenic ischemic stroke risk stratified by infection parameters.
Figure S12. Enrollment and infection frequency of cases and controls per month of the year.
Figure S13. Von Willebrand Factor and C-reactive protein in relation to time interval from infection or stroke onset to sample.
Figure S14. Von Willebrand Factor, C-reactive protein, factor VIII and fibrinogen in relation to baseline NIHSS score in cases.
Figure S15. Adjusted odds ratios (OR) and their confidence intervals (CI) for infection parameters and risk of early-onset cryptogenic ischemic stroke in cases not classified as 'likely atherothrombotic'.
Table S1. Description of risk-factor variables.
Table S2. Biomarker levels in included and excluded participants.
Table S3. Baseline biomarker levels in cases and controls.
Table S4. Demographics and comorbidities in early-onset cryptogenic ischemic stroke cases with and without preceding infection.
Table S5. Demographics and comorbidities in early-onset cryptogenic ischemic stroke cases with and without recent infection.
Table S6. Number and percentage of likely atherothrombotic cases in cases with and without infection characteristic.

Supplemental methods.

All coagulation measurements were performed at the accredited coagulation laboratory at the Helsinki University Hospital (Diagnostic Center, Helsinki, Finland). FVIII one-stage clotting assay (OSA) was performed with a silica-based activated partial thromboplastin time (APTT) reagent and FVIII-deficient plasma at a final dilution of 1:40. Von Willebrand factor (VWF) activity (IU/mL) (VWF:GPIbR) and antithrombin (AT) activity (%) were measured using specific reagents. Analyses were conducted on an automated coagulation analyzer. Plasma levels of functional fibrinogen (g/L) were measured by the Clauss method using a functional fibrinogen reagent. Protein C (PC) activity (%) was measured chromogenic substrate-based assay on an automated coagulation system. All the devices and reagents were verified according to the routine protocols in HUS Diagnostic Center and were in routine clinical use at the time of the study.

Figure S1. Questionnaire on preceding infections for cases and controls.

ID: ☐☐ ☐☐☐☐ ☐☐

Preceding Infections

Ask the patient (or proxy, if applicable) the following questions and, where necessary, verify information from primary care and hospital medical records.

1. **Think about the last 2 weeks immediately before your stroke. Did you have fever during that period?**

   ☐ No
   ☐ Yes, >37.5°C but <38.5°C (>99.5°F, <101.3°F)
   ☐ Yes, ≥38.5°C (≥101.3°F)
   ☐ Unknown

2. **Did you notice any symptoms of an infectious disease or has your doctor diagnosed an infectious disease within the 3-month period prior to onset of the stroke?**

   ☐ No → **Skip to question 12.**
   ☐ Yes
   ☐ Unknown → **Skip to question 12.**

3. **How was this infection confirmed?**

   ☐ No medical contact due to that infection
   ☐ Clinical examination by a physician
   ☐ Clinical examination with abnormal laboratory tests and/or radiologic findings
   ☐ Unknown

4. **Did you have fever (>37.5°C / >99.5°F) during that particular infection?**

   ☐ No
   ☐ Yes, >37.5°C but <38.5°C (>99.5°F, <101.3°F)
   ☐ Yes, ≥38.5°C (≥101.3°F)
   ☐ Unknown

5. **Choose the day or week of symptom onset of that infection (if >1 separate episodes, choose the most recent one)? Day 1 denotes the day immediately preceding the stroke:**

   ☐ Day 1 (yesterday)
   ☐ Days 2-3
   ☐ Days 4-7
   ☐ Week 2
   ☐ Week 3
   ☐ Week 4
   ☐ Weeks 5-8 (month 2)
   ☐ Weeks 9-12 (month 3)
   ☐ Unknown

6. **Describe the mode of the (most recent) infection:**

   ☐ Chronic with onset more than 4 weeks ago
   ☐ Acute onset
   ☐ Unknown

7. **Describe how the symptoms of the (most recent) infection correlate with onset of the qualifying stroke:**

   ☐ Symptoms had completely resolved at the time of stroke onset
   ☐ Symptoms were present at the time of stroke onset or hospital admission
   ☐ Unknown

Preceding Infections

8. During this infection, did you develop one or more of the following symptoms?

- ☐ Cough
- ☐ Purulent sputum
- ☐ Rhinitis
- ☐ Hoarseness
- ☐ Sore throat or problems with swallowing
- ☐ Diarrhea
- ☐ Dysuria
- ☐ Toothache
- ☐ Other symptoms → **What?**____
- ☐ Unknown

9. Did your doctor tell you whether the (most recent) infection was one of the following (*if no contact to doctor, indicate the most likely pathogen group*)?

- ☐ Bacterial
- ☐ Viral
- ☐ Fungal
- ☐ Parasitic
- ☐ Unknown

10. Did your doctor tell you whether the location of the (most recent) infection was one of the following (*if no contact to doctor, indicate the most likely location*)?

- ☐ Upper respiratory tract
- ☐ Lower respiratory tract
- ☐ Dental
- ☐ Urinary tract
- ☐ Gastrointestinal
- ☐ Skin
- ☐ Systemic
- ☐ Other → **What?**____
- ☐ Unknown

11. Was that infection treated with antimicrobial agents?

- ☐ No
- ☐ Yes, with antibiotics → **Specify below**
- ☐ Yes, with other anti-infectious drugs → **Specify below**
- ☐ Unknown

Name of the drug(s):____

12. Have you been vaccinated against influenza during the last season?

- ☐ No
- ☐ Yes
- ☐ Unknown

13. Have you been vaccinated against other diseases during the last season?

- ☐ No
- ☐ Yes → **Specify:**____
- ☐ Unknown

Preceding Infections

Ask the participant the following questions and, where necessary, verify information from primary care and hospital medical records.

1. Think about the last 2 weeks immediately before this interview. Did you have fever during that period?

☐ No
☐ Yes, >37.5°C but <38.5°C (>99.5°F, <101.3°F)
☐ Yes, ≥38.5°C (≥101.3°F)
☐ Unknown

2. Did you notice any symptoms of an infectious disease or have your doctor diagnosed an infectious disease within the 3-month period prior to this interview?

☐ No → **Skip to question 11.**
☐ Yes
☐ Unknown → **Skip to question 11.**

3. How was this infection confirmed?

☐ No medical contact due to that infection
☐ Clinical examination by a physician
☐ Clinical examination with abnormal laboratory tests and/or radiologic findings
☐ Unknown

4. Did you have fever (>37.5°C / >99.5°F) during that particular infection?

☐ No
☐ Yes, >37.5°C but <38.5°C (>99.5°F, <101.3°F)
☐ Yes, ≥38.5°C (≥101.3°F)
☐ Unknown

5. Choose the day or week of symptom onset of that infection (if >1 separate episodes, choose the most recent one)? Day 1 denotes the day immediately preceding this interview.

☐ Day 1 (yesterday)
☐ Days 2-3
☐ Days 4-7
☐ Week 2
☐ Week 3
☐ Week 4
☐ Weeks 5-8 (2 months ago)
☐ Weeks 9-12 (3 months ago)
☐ Unknown

6. Describe the mode of the (most recent) infection:

☐ Chronic with onset more than 4 weeks ago
☐ Acute onset
☐ Unknown

Preceding Infections

7. During this infection did you develop one or more of the following symptoms?

- ☐ Cough
- ☐ Purulent sputum
- ☐ Rhinitis
- ☐ Hoarseness
- ☐ Sore throat or problems with swallowing
- ☐ Diarrhea
- ☐ Dysuria
- ☐ Toothache
- ☐ Other symptoms → **What?**____
- ☐ Unknown

8. Did your doctor tell you whether the (most recent) infection was one of the following (*if no contact to doctor, indicate the most likely pathogen group*)?

- ☐ Bacterial
- ☐ Viral
- ☐ Fungal
- ☐ Parasitic
- ☐ Unknown

9. Did your doctor tell you whether the location of the (most recent) infection was one of the following (*if no contact to doctor, indicate the most likely location*)?

- ☐ Upper respiratory tract
- ☐ Lower respiratory tract
- ☐ Dental
- ☐ Urinary tract
- ☐ Gastrointestinal
- ☐ Skin
- ☐ Systemic
- ☐ Other → **What?**____
- ☐ Unknown

10. Was that infection treated with antimicrobial agents?

- ☐ No
- ☐ Yes, with antibiotics → **Specify below**
- ☐ Yes, with other anti-infectious drugs → **Specify below**
- ☐ Unknown

Name of the drug(s):____

11. Have you been vaccinated against influenza during the last season?

- ☐ No
- ☐ Yes
- ☐ Unknown

12. Have you been vaccinated against other diseases during the last season?

- ☐ No
- ☐ Yes → **Specify:**____
- ☐ Unknown

Figure S2. Unadjusted odds ratios (OR) and their confidence intervals (CI) for infection variables and risk of early-onset cryptogenic ischemic stroke.

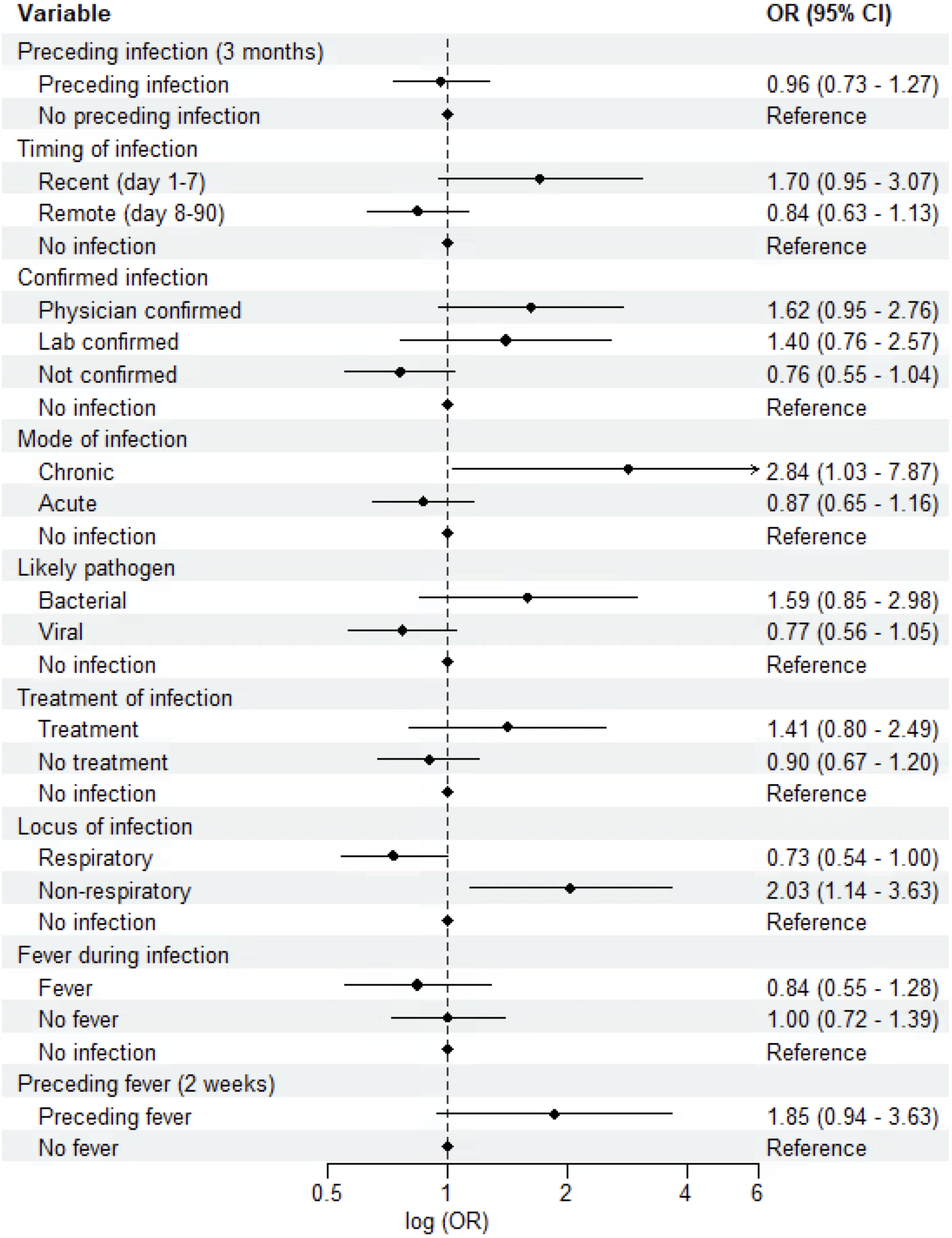

Data are presented as odds ratio (OR) and 95% confidence interval (CI). Dotted line = reference line. Chronic = duration > 4 weeks.

Figure S3. Distribution of von Willebrand Factor, Factor VIII and fibrinogen by preceding infection and mode of infection.

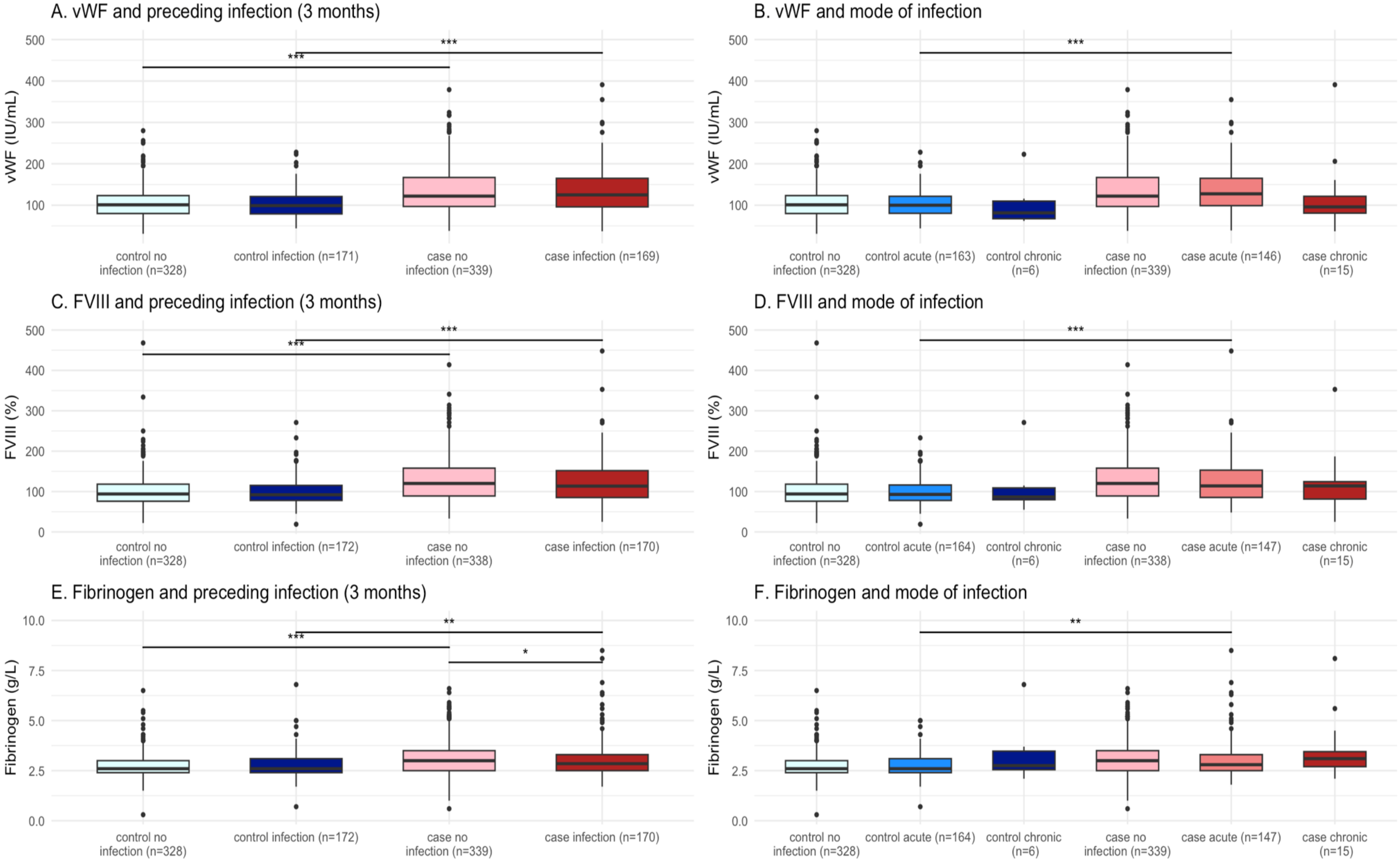

Data are presented as median, with interquarrtile ranges, and confidence intervals. vWF = von Willebrand Factor, FVIII = factor VIII, recent = within 1-7 days before stroke/study visit, remote = within 8-90 days before stroke/study visit. Blue = control, red = case. “*”= p< 0.05, “**”= p<0.01, “***”= p<0.001. Only significant comparisons shown.

Figure S4. Distribution of C-reactive protein by preceding infection and mode of infection.

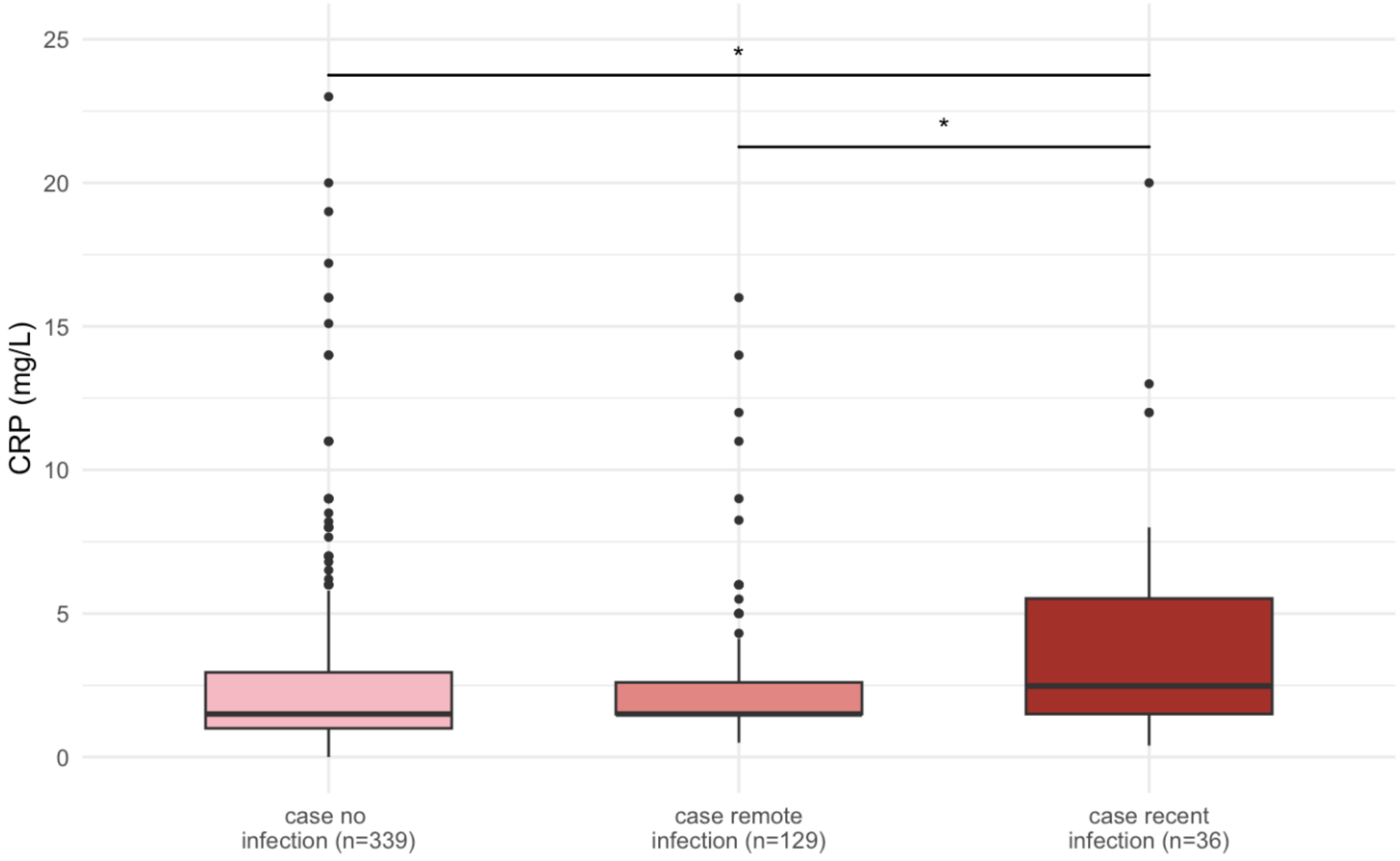

Data are presented as median, with interquarrtile ranges, and confidence intervals. Recent = within 1-7 days before stroke/study visit, remote = within 8-90 days before stroke/study visit. "*"= p< 0.05, "**"= p<0.01, "***"= p<0.001. Only significant comparisons shown.

Figure S5. Antithrombin III (AT3) levels across infection parameters.

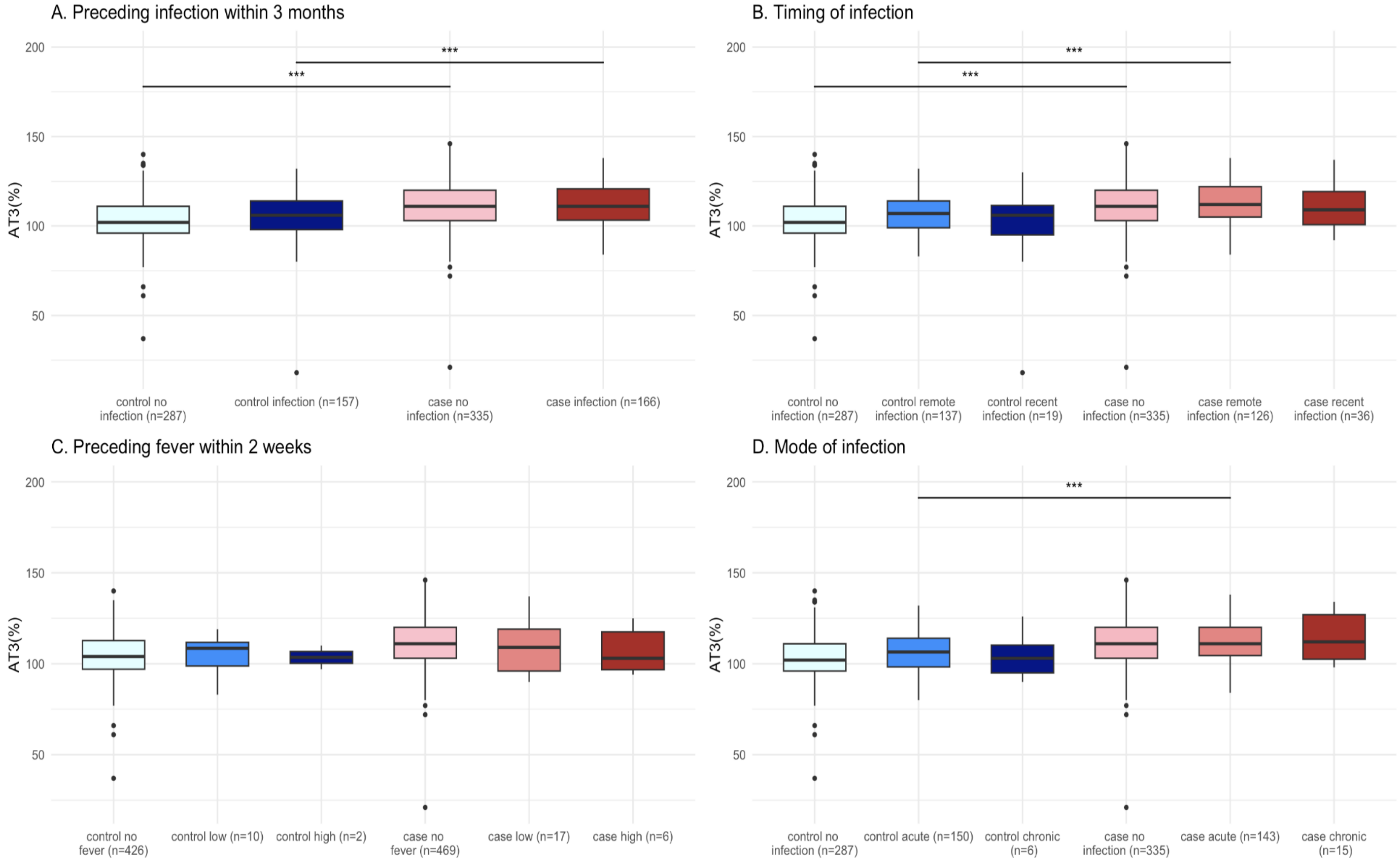

Data are presented as median, with interquarrtile ranges, and confidence intervals. AT3 = antithrombin III, recent = within 1-7 days before stroke/study visit, remote = within 8-90 days before stroke/study visit, fever low = body temperature between 37.5°C and 38.5°C, fever high = body temperature > 38.5°C. Blue = control, red = case. "*"= p< 0.05, "**"= p<0.01, "***"= p<0.001. Only significant comparisons shown.

Figure S6. Protein C (PC) levels across infection parameters.

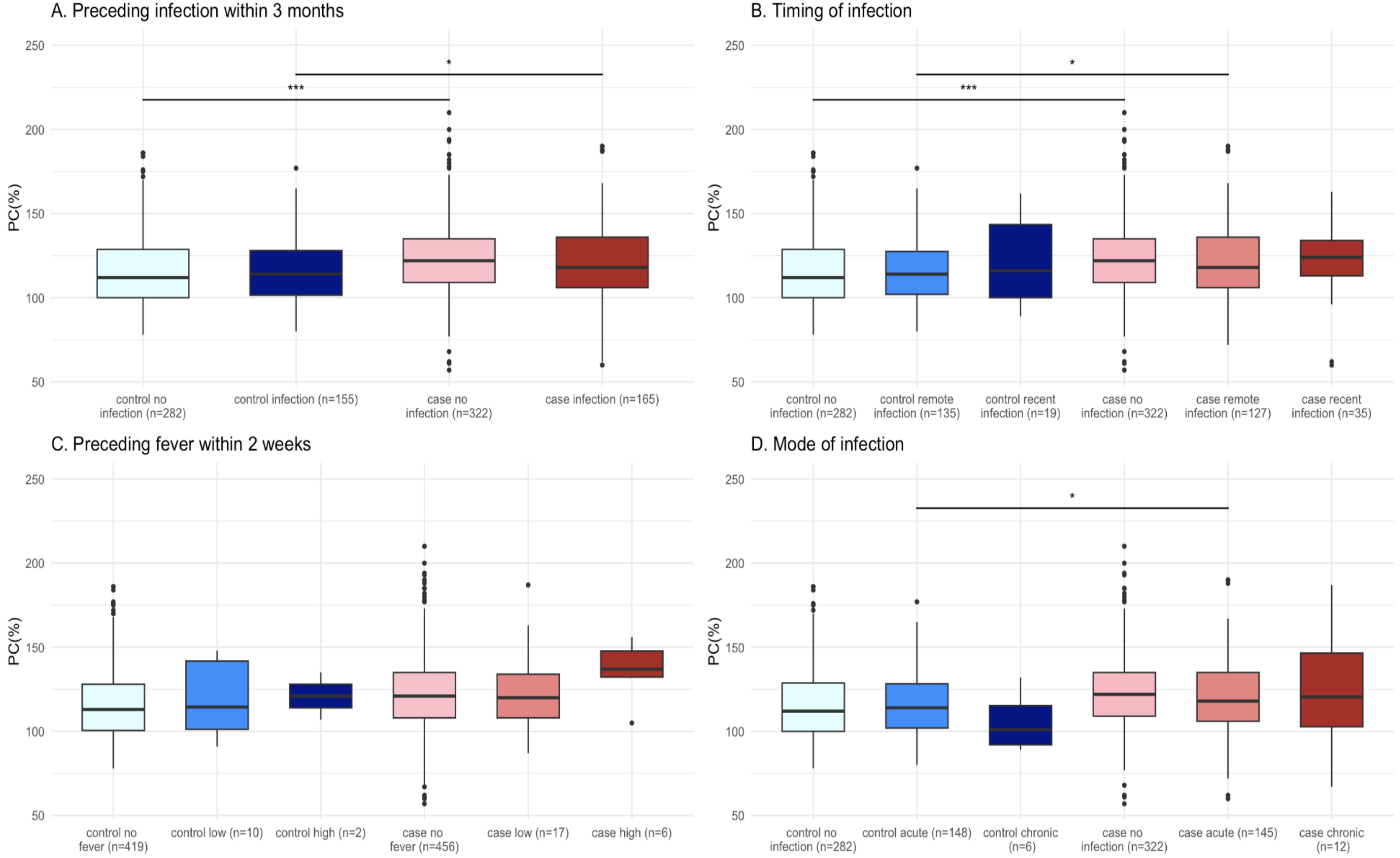

Data are presented as median, with interquarrtile ranges, and confidence intervals. PC = protein C, recent = within 1-7 days before stroke/study visit, remote = within 8-90 days before stroke/study visit, fever low = body temperature between 37.5°C and 38.5°C, fever high = body temperature > 38.5°C. Blue = control, red = case. “*”= p< 0.05, “**”= p<0.01, “***”= p<0.001. Only significant comparisons shown.

Figure S7. Distribution of von Willebrand Factor, Factor VIII, fibrinogen and antithrombin III by level of preceding fever in the preceding two weeks in cases only.

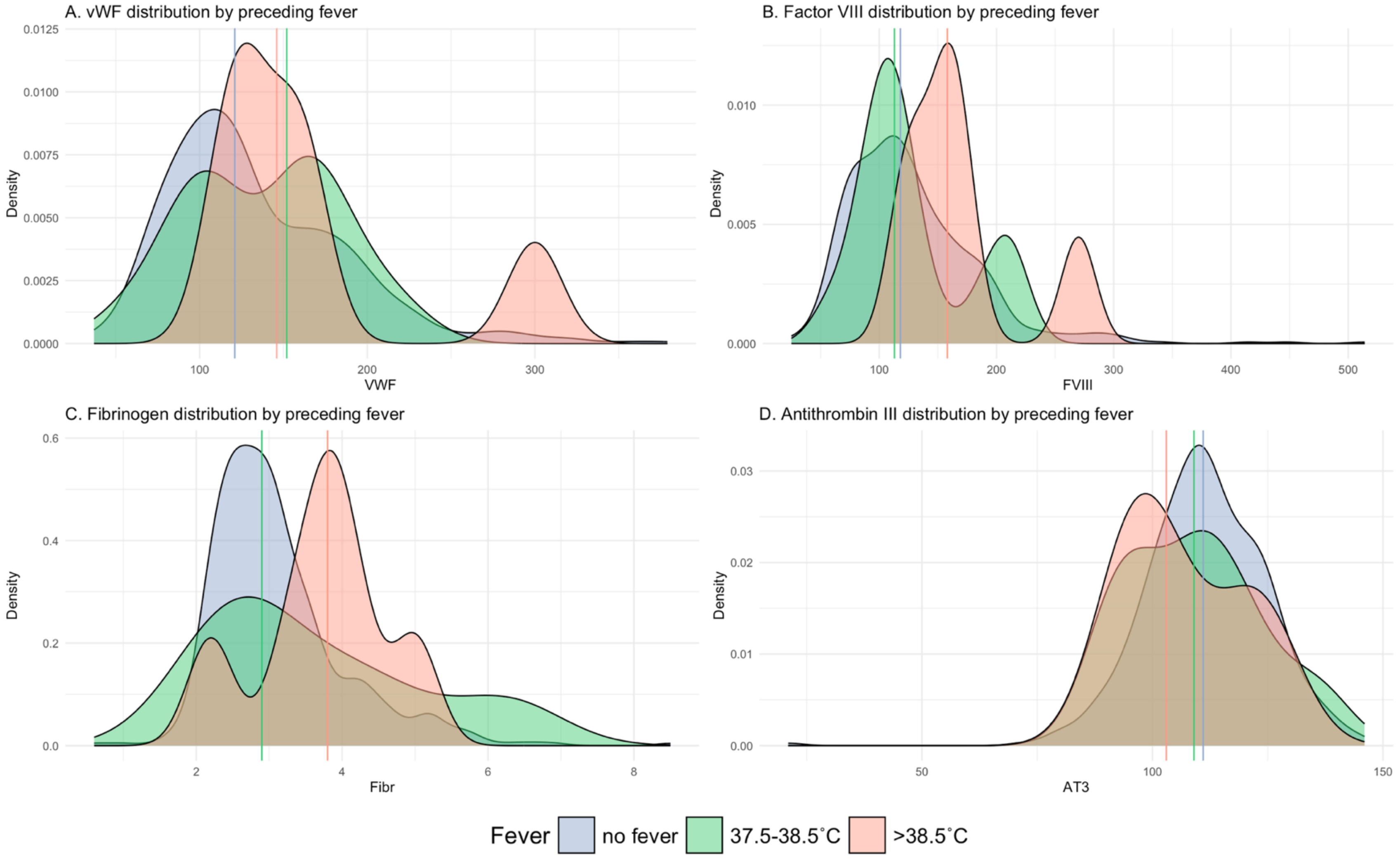

Data are show as density plots with medians. Fever = elevated body temperature in the past two weeks. Gray = no fever, green = body temperature 37.5-38.5°C, red = body temperature > 38.5°C. Median colors correspond to the density plot.

Figure S8. Von Willebrand Factor levels across infection parameters after three months.

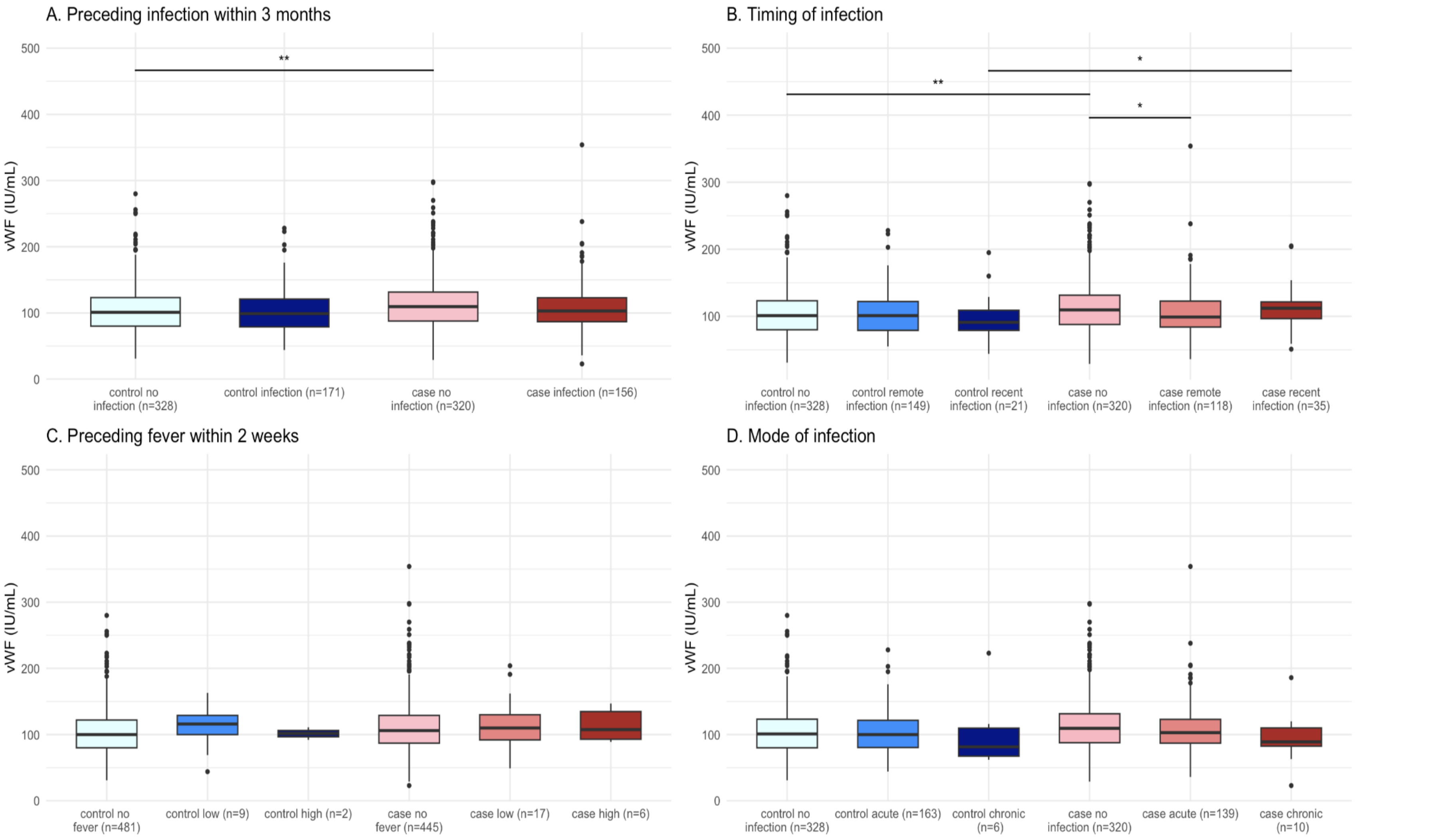

Data are presented as median, with interquarrtile ranges, and confidence intervals. vWF = von Willebrand Factor, recent = within 1-7 days before stroke/study visit, remote = within 8-90 days before stroke/study visit, fever low = body temperature between 37.5°C and 38.5°C, fever high = body temperature > 38.5°C. Blue = control, red = case. "*"= p< 0.05, "**"= p<0.01, "***"= p<0.001. Only significant comparisons shown.

Figure S9. Coagulation factor VIII levels across infection parameters after three months.

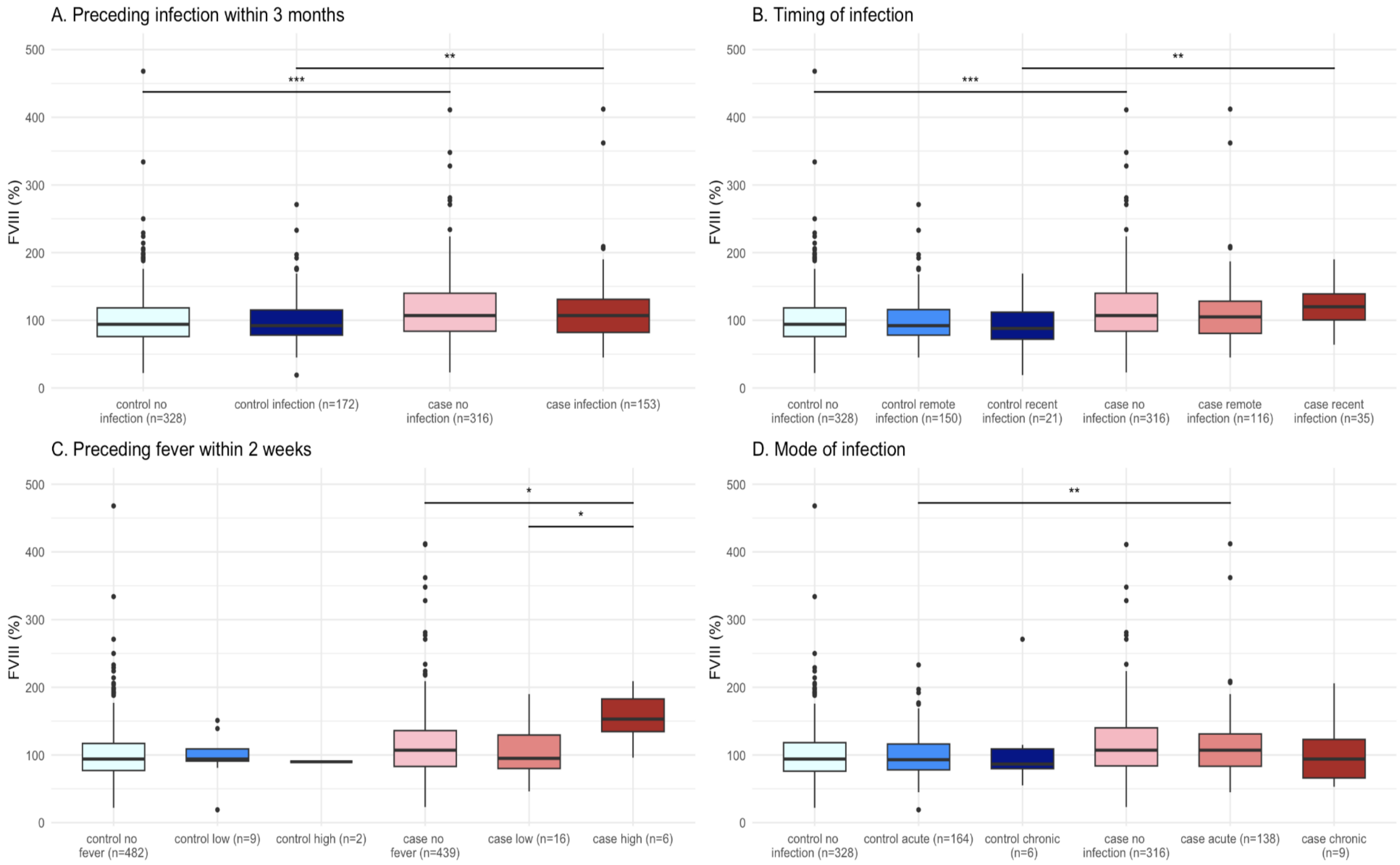

Data are presented as median, with interquarrtile ranges, and confidence intervals. FVIII = factor VIII, recent = within 1-7 days before stroke/study visit, remote = within 8-90 days before stroke/study visit, fever low = body temperature between 37.5°C and 38.5°C, fever high = body temperature > 38.5°C. Blue = control, red = case. "*"= p< 0.05, "**"= p<0.01, "***"= p<0.001. Only significant comparisons shown.

Figure S10. Fibrinogen levels across infection parameters after three months.

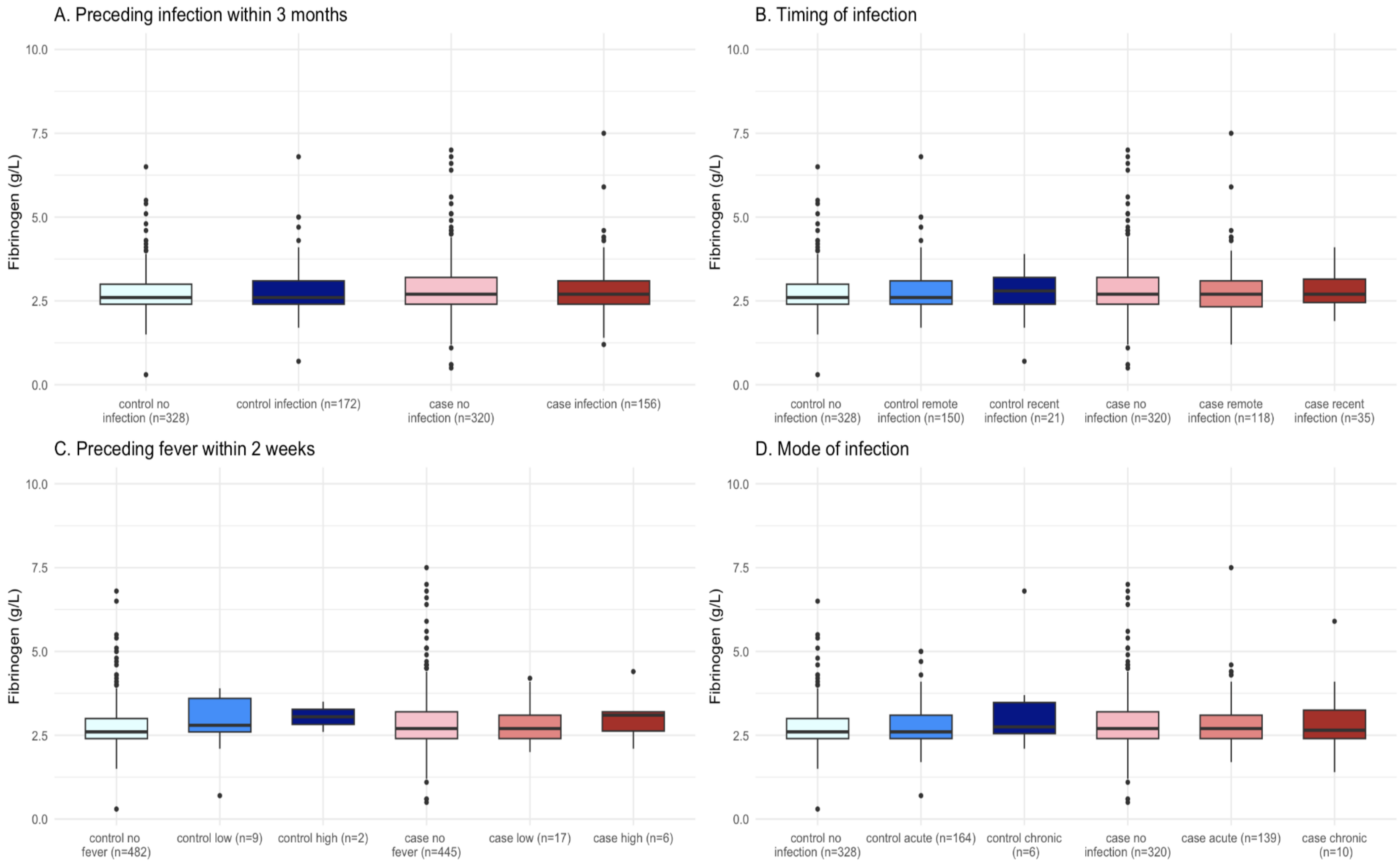

Data are presented as median, with interquarrtile ranges, and confidence intervals. Recent = within 1-7 days before stroke/study visit, remote = within 8-90 days before stroke/study visit, fever low = body temperature between 37.5°C and 38.5°C, fever high = body temperature > 38.5°C. Blue = control, red = case. "*"= p< 0.05, "**"= p<0.01, "***"= p<0.001. Only significant comparisons shown.

Figure S11. Unadjusted odds ratios and 95% confidence intervals of von Willebrand Factor, coagulation factor VIII and fibrinogen per standard deviation increase, and cryptogenic ischemic stroke risk stratified by infection parameters.

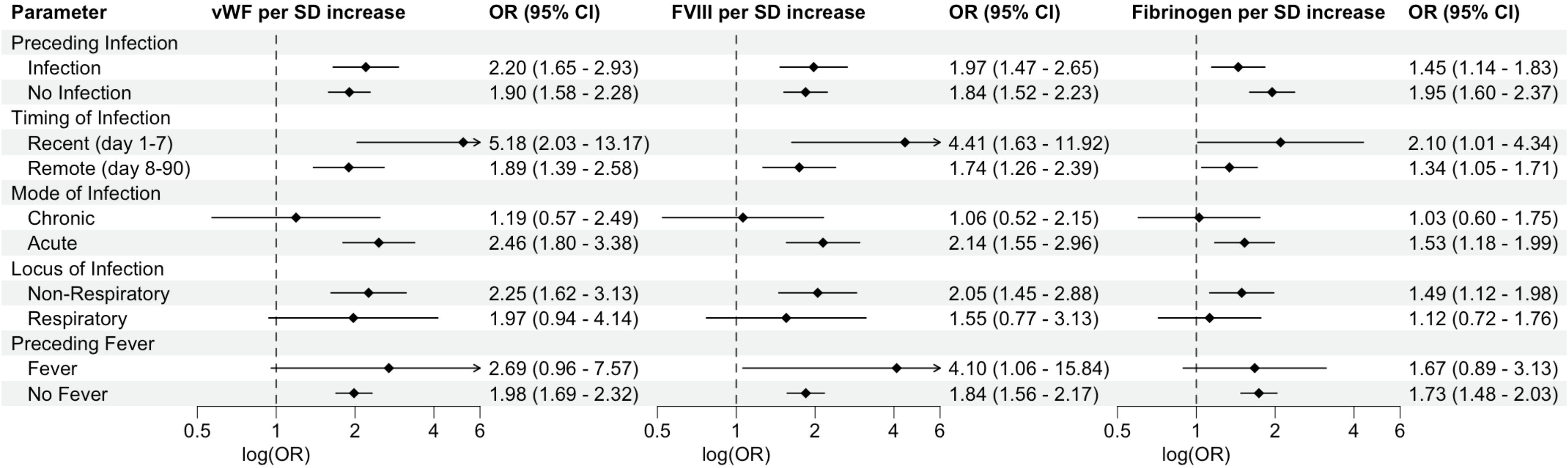

Parameter	vWF per SD increase OR (95% CI)	FVIII per SD increase OR (95% CI)	Fibrinogen per SD increase OR (95% CI)
Preceding Infection			
Infection	2.20 (1.65 - 2.93)	1.97 (1.47 - 2.65)	1.45 (1.14 - 1.83)
No Infection	1.90 (1.58 - 2.28)	1.84 (1.52 - 2.23)	1.95 (1.60 - 2.37)
Timing of Infection			
Recent (day 1-7)	5.18 (2.03 - 13.17)	4.41 (1.63 - 11.92)	2.10 (1.01 - 4.34)
Remote (day 8-90)	1.89 (1.39 - 2.58)	1.74 (1.26 - 2.39)	1.34 (1.05 - 1.71)
Mode of Infection			
Chronic	1.19 (0.57 - 2.49)	1.06 (0.52 - 2.15)	1.03 (0.60 - 1.75)
Acute	2.46 (1.80 - 3.38)	2.14 (1.55 - 2.96)	1.53 (1.18 - 1.99)
Locus of Infection			
Non-Respiratory	2.25 (1.62 - 3.13)	2.05 (1.45 - 2.88)	1.49 (1.12 - 1.98)
Respiratory	1.97 (0.94 - 4.14)	1.55 (0.77 - 3.13)	1.12 (0.72 - 1.76)
Preceding Fever			
Fever	2.69 (0.96 - 7.57)	4.10 (1.06 - 15.84)	1.67 (0.89 - 3.13)
No Fever	1.98 (1.69 - 2.32)	1.84 (1.56 - 2.17)	1.73 (1.48 - 2.03)

Data are shown as odds ratio (OR) and 95% confidence interval (CI). SD = standard deviation, vWF = von Willebrand Factor, FVIII = factor VIII. Dotted line = reference line. Preceding infection = any infection in the past three months, preceding fever = body temperature > 37.5 degrees Celcius in the past two weeks, chronic = duration > four weeks.

Figure S12. Enrollment and infection frequency of cases and controls per month of the year.

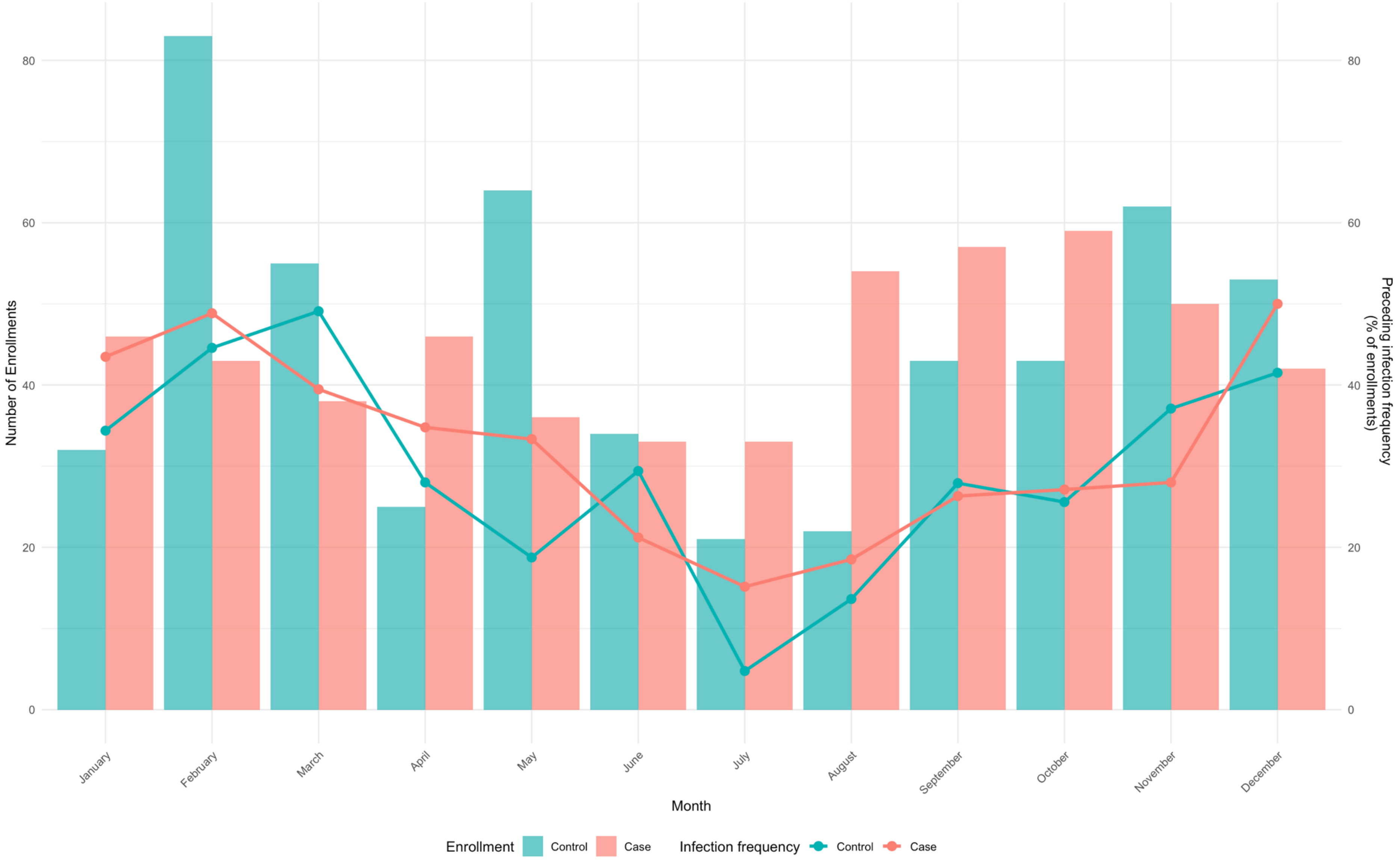

Figure S13. Von Willebrand Factor and C-reactive protein in relation to time interval from infection or stroke onset to sample.

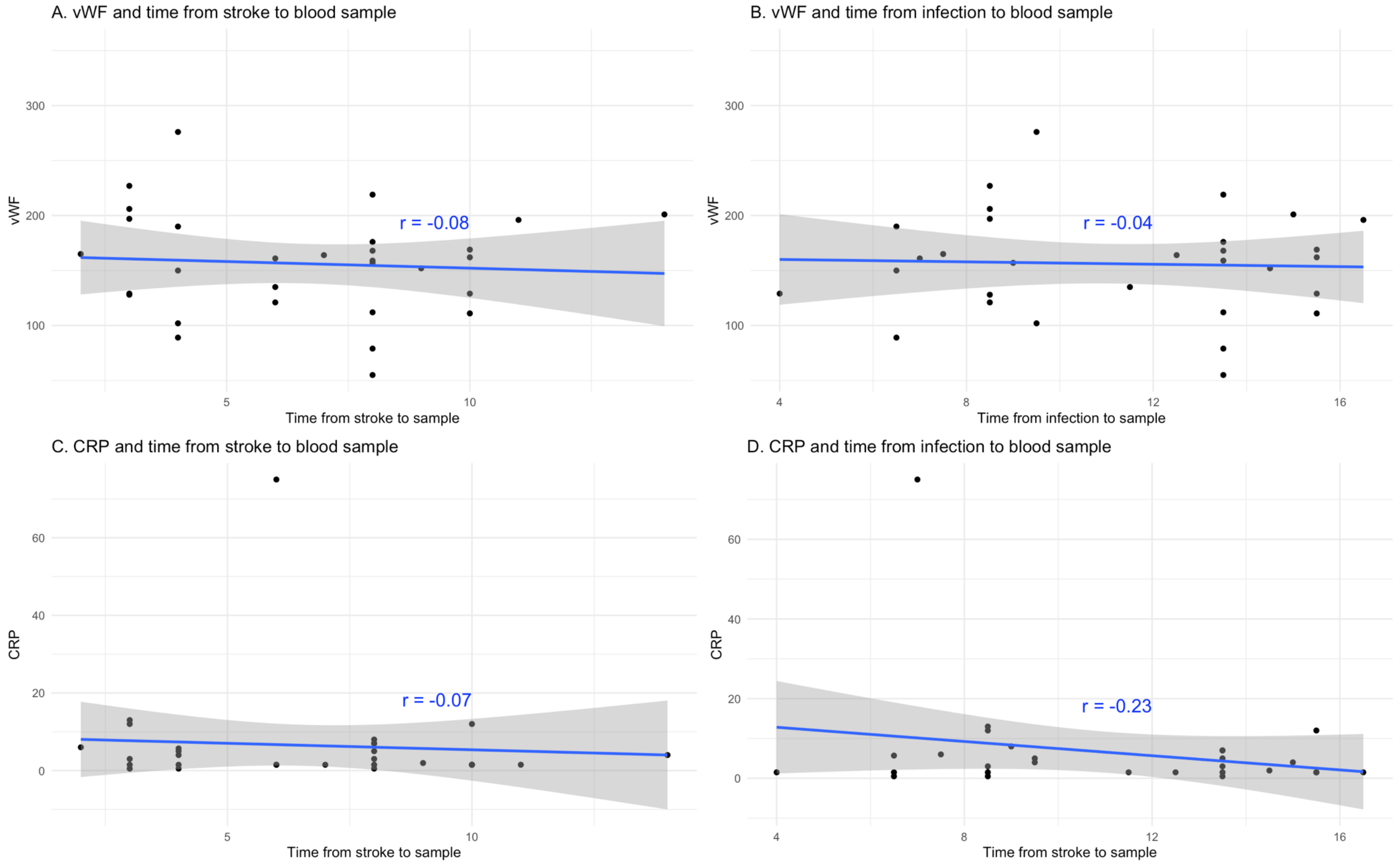

Data are shown as scatterplot with regression line and corresponding confidence interval. R = correlation coefficient, vWF = von Willebrand Factor, CRP = C-Reactive Protein, time in days.

Figure S14. Von Willebrand Factor, C-reactive protein, factor VIII and fibrinogen in relation to baseline NIHSS score in cases.

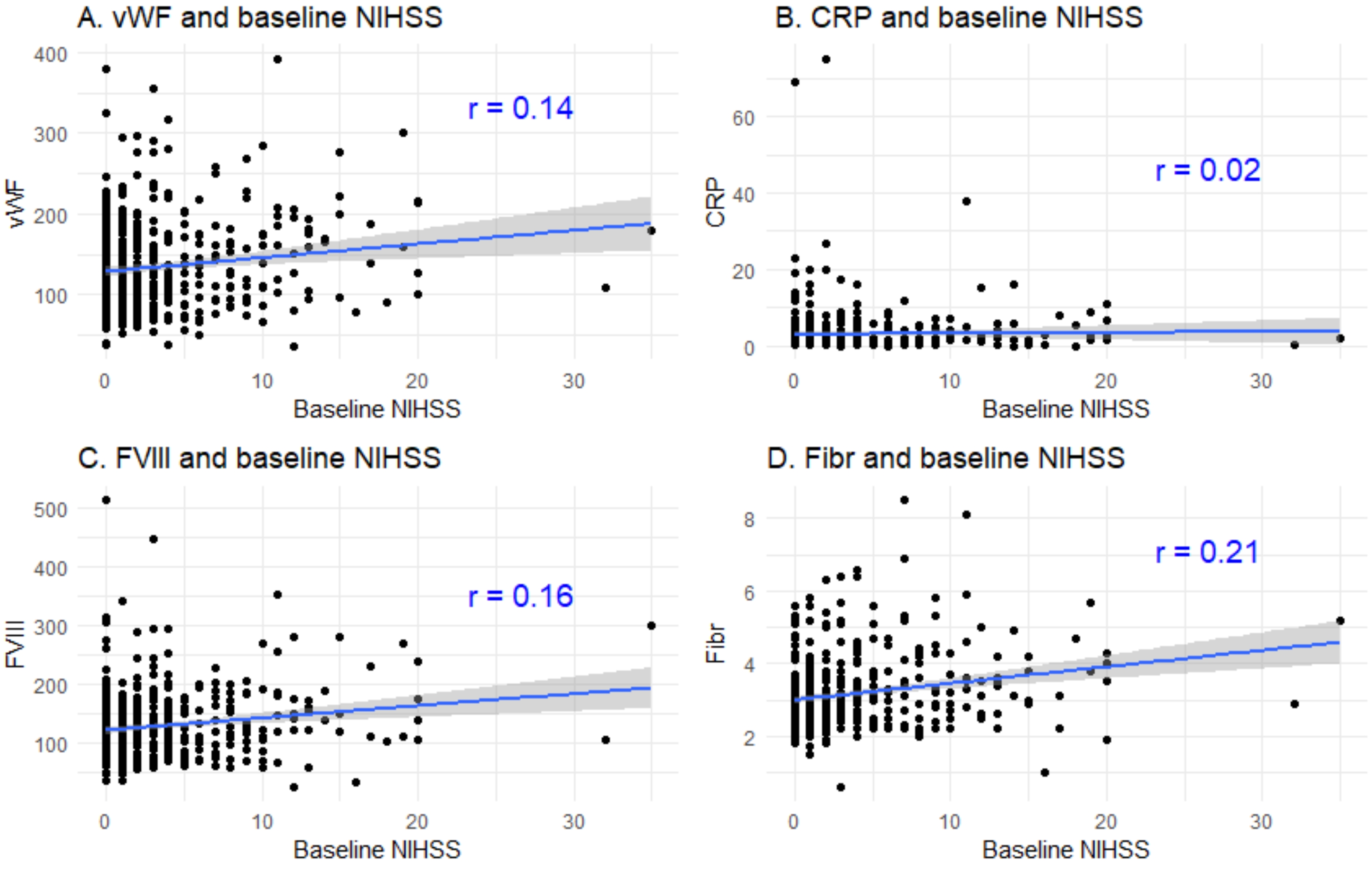

Data are shown as scatterplot with regression line and corresponding confidence interval. R = correlation coefficient, vWF = von Willebrand Factor, CRP = C-Reactive Protein, FVIII = Factor VIII, Fibr = Fibrinogen, NIHSS = National Institute of Health Stroke Scale.

Figure S15. Adjusted odds ratios (OR) and their confidence intervals (CI) for infection parameters and risk of early-onset cryptogenic ischemic stroke in cases not classified as 'likely atherothrombotic'.

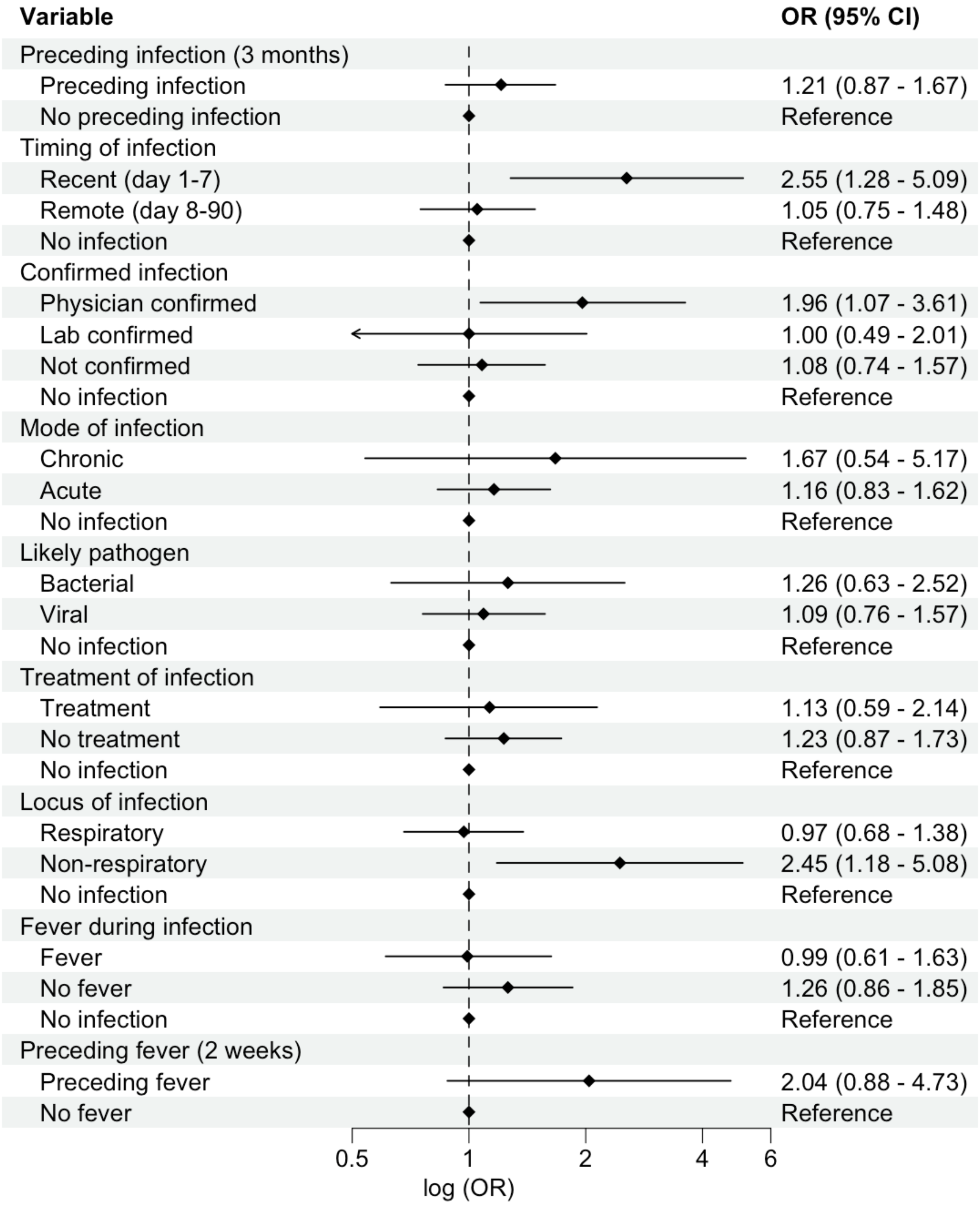

Data are presented as odds ratio (OR) and 95% confidence interval (CI). Dotted line = reference line. Chronic = duration > 4 weeks.

Table S1. Description of risk-factor variables.

Variable	Definition
Low level of education	Either primary or lower or upper secondary education. Structured questionnaire.
History of cardiovascular disease	History of coronary heart disease, congestive heart failure, peripheral arterial disease, arterial thrombosis, aneurysm, aortic or valvular disease. Patient history and medical records.
Diabetes mellitus	Diagnosis and/or antidiabetic medication. Patient history and medical records.
Hypertension	Diagnosis and/or antihypertensive medication or mean of two blood pressure measurements over 140/90 at study visit. Patient history and medical records.
Hypercholesterolemia	Diagnosis and/or antilipemic medication. Patient history and medical records.
Current tobacco smoking	Smoking an average of at least one cigarette a day. Structured questionnaire.
Abdominal obesity	Waist-to-hip-ratio >0.85 in men or >0.95 in women.
History of chronic multisystem disorder	History of any of inflammatory bowel disease, any other autoimmune disease, chronic kidney/liver disease, or hematologic disease or known thrombophilia. Patient history and medical records.
History of venous thrombosis	Patient history and medical records.
History of malignancy	History of any malignancy based on comorbidities and VTE risk factors. Patient history and medical records.
Migraine with aura	Based on a validated migraine screener.
Current illicit drug use	Any illicit drug use within the past 12 months. Structured questionnaire
Current estrogen use	Any pre-stroke estrogen preparate use, any route of administration. Patient history and medical records.

Table S2. Biomarker levels in included and excluded participants.

Biomarker	Excluded participants (n=18)	Included participants (n = 1,074)	P-value
von Willebrand Factor (IU/mL)	105 (80-125)	110 (86-146)	0.443
Factor VIII (%)	89 (72-112)	105 (81-136)	0.218
Fibrinogen (g/L)	2.65 (2.18-3.53)	2.8 (2.4-3.2)	0.583
Antithrombin III (%)	109 (104-115)	108 (99-116)	0.562
Protein C (%)	115.5 (109.8-127.5)	117 (104-132)	0.822
C-reactive protein (mg/L)	1.53 (1.50-3.63)	1.5 (1.5-3.0)	0.476

Data are median (interquartile range).

Table S3. Baseline biomarker levels in cases and controls.

Biomarker	N	Cases (n = 537)	Controls (n = 537)	P-value
von Willebrand Factor (IU/mL)	1,007	122 (96-165)	100 (80-122)	<0.001
Factor VIII (%)	1,008	118 (89-156)	94 (77-117)	<0.001
Fibrinogen (g/L)	1,009	2.9 (2.5-3.5)	2.6 (2.4-3.0)	<0.001
Antithrombin III (%)	945	111 (103-120)	104 (97-112)	<0.001
Protein C (%)	924	121 (108-135)	113 (101-128)	<0.001
C-reactive protein (mg/L)	508	1.5 (1.5-3)	NA	NA

Data are median (interquartile range).

Table S4. Demographics and comorbidities in early-onset cryptogenic ischemic stroke cases with and without preceding infection.

**Characteristic**	**Valid N**	**Preceding infection (N=172)**	**No preceding infection (N=365)**	***P***
**Demographics**				
Age, years	537	40.3 (33.8-45.6)	41.0 (34.3-45.9)	0.666
18-39 years	537	82 (47.7)	163 (44.7)	0.513
40-49 years	537	90 (55.3)	202 (52.3)	0.513
Female sex	537	78 (45.3)	176 (48.2)	0.534
Low level of education	535	208 (57.3)	88 (51.2)	0.182
**Traditional risk factors**				
Cardiovascular disease	537	4 (2.3)	11 (3)	0.784
Diabetes	537	3 (1.7)	12 (3.3)	0.407
Hypercholesterolemia	537	5 (2.9)	7 (1.9)	0.535
Hypertension	537	54 (31.4)	132 (36.2)	0.278
Current smoking	534	45 (26.2)	129 (35.6)	**0.029**
Abdominal obesity	537	98 (57.0)	219 (60.0)	0.506
No. of likely atherothrombotic	534	14 (8.1)	60 (16.6)	**0.008**
**Non-traditional risk factors**				
Chronic multisystem disorder	536	26 (15.2)	51 (14.0)	0.705
History of venous thrombosis	535	9 (5.2)	9 (2.5)	0.099
History of malignancy	537	3 (1.7)	7 (1.9)	>.999
Migraine with aura	537	81 (47.1)	143 (39.2)	0.083
Current illicit drug use	537	11 (6.4)	30 (8.2)	0.458
Current estrogen use	254	18 (23.1)	50 (28.4)	0.376
**Interval from stroke to blood sample**				
Days from stroke to blood sample	406	7 (4-9)	6 (4-9)	0.205
**National Institutes of Health Stroke Scale (NIHSS)**				
NIHSS-score	534	2 (0-4)	2 (1-5)	0.150

Data are median (interquartile range) for continuous and n (%) for categorical variables.

Table S5. Demographics and comorbidities in early-onset cryptogenic ischemic stroke cases with and without recent infection.

Characteristic	Valid N	Recent infection (N=37)	No recent infection (N=496)	*P*
**Demographics**				
Age, years	503	40.9 (34.1-46.0)	40.8 (34.1-45.7)	0.783
18-39 years	533	17 (45.9)	227 (45.8)	0.983
40-49 years	533	20 (54.1)	269 (54.2)	0.983
Female sex	533	21 (56.8)	231 (46.6)	0.231
Low level of education	531	19 (51.4)	275 (55.7)	0.610
**Traditional risk factors**				
Cardiovascular disease	533	1 (2.7)	14 (2.8)	>0.999
Diabetes	533	0 (0)	15 (3.0)	0.614
Hypercholesterolemia	533	1 (2.7)	11 (2.2)	0.582
Hypertension	533	13 (35.1)	172 (34.7)	0.955
Current smoking	530	9 (24.3)	164 (33.3)	0.263
Abdominal obesity	533	21 (56.8)	294 (59.3)	0.764
No. of likely atherothrombotic	530	4 (10.8)	70 (14.2)	0.566
**Non-traditional risk factors**				
Chronic multisystem disorder	532	5 (13.9)	71 (14.3)	0.944
History of venous thrombosis	531	4 (10.8)	14 (2.8)	**0.030**
History of malignancy	533	0 (0)	10 (2.0)	>0.999
Migraine with aura	533	16 (43.2)	207 (41.7)	0.858
Current illicit drug use	533	3 (8.1)	38 (7.7)	0.757
Current estrogen use	252	5 (23.8)	62 (26.8)	0.763
**Interval from stroke to blood sample**				
Days from stroke to blood sample	403	7.5 (4-8)	6 (4-9)	0.904
**National Institutes of Health Stroke Scale (NIHSS)**				
NIHSS-score	530	1.5 (0-2.2)	2 (1-4)	0.107

Data are median (interquartile range) for continuous and n (%) for categorical variables.

Table S6. Number and percentage of likely atherothrombotic cases in cases with and without infection characteristic.

Variable	N	N(%) in cases without infection characteristic	N(%) in cases with infection characteristic	P-value
**Confirmed infection**				
Confirmed	172	7 (6.7)	7 (10.4)	0.377
By physician	172	12 (9.0)	2 (5.1)	0.739
By laboratory results	172	9 (6.2)	5 (17.9)	0.055
**Mode of infection**				
Chronic (yes) vs. acute (no)	164	4 (26.7)	10 (6.7)	**0.027**
**Likely pathogen**				
Viral (yes) vs. bacterial (no)	140	3 (9.7)	6 (5.5)	0.415
**Treatment of infection**				
Treatment received	171	10 (7.2)	4 (12.1)	0.477
**Locus of infection**				
Non-respiratory (yes) vs, respiratory (no)	157	7 (6.0)	5 (12.5)	0.184
**Fever during infection**				
Fever during infection	157	10 (9.3)	4 (8.2)	>0.999

Data are n(%).
