# Supplementary material for: Preceding Infections and Coagulation Biomarkers in Early-Onset Cryptogenic Ischemic Stroke
Source: Stroke. 2026 Mar 17;57(5):1220–30. doi: 10.1161/STROKEAHA.125.052134 (PMC13117575; doi:10.1161/STROKEAHA.125.052134)
Supplement: Supplementary file 2 [file str-57-1220-s002.pdf]

STROBE Statement—checklist of items that should be included in reports of observational studies

	Item No.	Recommendation	Page No.	Relevant text from manuscript
**Title and abstract**	1	(*a*) Indicate the study's design with a commonly used term in the title or the abstract	4	Line 65
		(*b*) Provide in the abstract an informative and balanced summary of what was done and what was found	4	Line 59-84
**Introduction**				
Background/rationale	2	Explain the scientific background and rationale for the investigation being reported	6-7	Line 102-127
Objectives	3	State specific objectives, including any prespecified hypotheses	7	Line 128-129
**Methods**				
Study design	4	Present key elements of study design early in the paper	8-9	Line 131-157
Setting	5	Describe the setting, locations, and relevant dates, including periods of recruitment, exposure, follow-up, and data collection	8-9	Line 131-155
Participants	6	(*a*) *Cohort study*—Give the eligibility criteria, and the sources and methods of selection of participants. Describe methods of follow-up<br>*Case-control study*—Give the eligibility criteria, and the sources and methods of case ascertainment and control selection. Give the rationale for the choice of cases and controls<br>*Cross-sectional study*—Give the eligibility criteria, and the sources and methods of selection of participants	8-9	Line 131-155
		(*b*) *Cohort study*—For matched studies, give matching criteria and number of exposed and unexposed<br>*Case-control study*—For matched studies, give matching criteria and the number of controls per case	8	Line 142-144
Variables	7	Clearly define all outcomes, exposures, predictors, potential confounders, and effect modifiers. Give diagnostic criteria, if applicable	9-10	Line 158-186
Data sources/ measurement	8*	For each variable of interest, give sources of data and details of methods of assessment (measurement). Describe comparability of assessment methods if there is more than one group	9-10	Line 158-186
Bias	9	Describe any efforts to address potential sources of bias	10-11	Line 188-226
Study size	10	Explain how the study size was arrived at	8	Described previously (line 138-139)

Continued on next page

Quantitative variables	11	Explain how quantitative variables were handled in the analyses. If applicable, describe which groupings were chosen and why	9-11	Line 158-226
Statistical methods	12	(*a*) Describe all statistical methods, including those used to control for confounding	10-11	Line 188-226
		(*b*) Describe any methods used to examine subgroups and interactions	9 11	Line 212-226
		(*c*) Explain how missing data were addressed	25-28	Valid N mentioned in tables and figures
		(*d*) *Cohort study*—If applicable, explain how loss to follow-up was addressed<br>*Case-control study*—If applicable, explain how matching of cases and controls was addressed<br>*Cross-sectional study*—If applicable, describe analytical methods taking account of sampling strategy	8-11	Line 142-144, 188-226
		(*e*) Describe any sensitivity analyses	11	Line 212-226
**Results**				
Participants	13*	(a) Report numbers of individuals at each stage of study—eg numbers potentially eligible, examined for eligibility, confirmed eligible, included in the study, completing follow-up, and analysed	13	Line 231-233
		(b) Give reasons for non-participation at each stage	13	Line 231-233
		(c) Consider use of a flow diagram	N/A	
Descriptive data	14*	(a) Give characteristics of study participants (eg demographic, clinical, social) and information on exposures and potential confounders	13-14	Line 231-255
		(b) Indicate number of participants with missing data for each variable of interest	25-27	Table 1 and 2
		(c) *Cohort study*—Summarise follow-up time (eg, average and total amount)	N/A	
Outcome data	15*	*Cohort study*—Report numbers of outcome events or summary measures over time	N/A	
		Case-control study—Report numbers in each exposure category, or summary measures of exposure	26-27	Table 2
		Cross-sectional study—Report numbers of outcome events or summary measures	N/A	
Main results	16	(*a*) Give unadjusted estimates and, if applicable, confounder-adjusted estimates and their precision (eg, 95% confidence interval). Make clear which confounders were adjusted for and why they were included	14	Line 256-262, Unadjusted ORs can be found in Figure S2
		(*b*) Report category boundaries when continuous variables were categorized	9	Fever was defined as a body temperature exceeding 37.5°C (line 174-176)
		(*c*) If relevant, consider translating estimates of relative risk into absolute risk for a meaningful time period	N/A	

Continued on next page

Other analyses	17	Report other analyses done—eg analyses of subgroups and interactions, and sensitivity analyses	15-16	Line 292-303
**Discussion**				
Key results	18	Summarise key results with reference to study objectives	17	Line 305-313
Limitations	19	Discuss limitations of the study, taking into account sources of potential bias or imprecision. Discuss both direction and magnitude of any potential bias	19-20	Line 369-388
Interpretation	20	Give a cautious overall interpretation of results considering objectives, limitations, multiplicity of analyses, results from similar studies, and other relevant evidence	20	Line 389-396
Generalisability	21	Discuss the generalisability (external validity) of the study results	19	Line 369-370
**Other information**				
Funding	22	Give the source of funding and the role of the funders for the present study and, if applicable, for the original study on which the present article is based	21	Line 403-406

*Give information separately for cases and controls in case-control studies and, if applicable, for exposed and unexposed groups in cohort and cross-sectional studies.

Note: An Explanation and Elaboration article discusses each checklist item and gives methodological background and published examples of transparent reporting. The STROBE checklist is best used in conjunction with this article (freely available on the Web sites of PLoS Medicine at http://www.plosmedicine.org/, Annals of Internal Medicine at http://www.annals.org/, and Epidemiology at http://www.epidem.com/). Information on the STROBE Initiative is available at www.strobe-statement.org.
